# Pheromones that correlate with reproductive success in competitive conditions

**DOI:** 10.1038/s41598-021-01507-9

**Published:** 2021-11-09

**Authors:** Kenneth C. Luzynski, Doris Nicolakis, Maria Adelaide Marconi, Sarah M. Zala, Jae Kwak, Dustin J. Penn

**Affiliations:** 1 Department of Interdisciplinary Life Sciences, Konrad Lorenz Institute of Ethology, University of Veterinary Medicine Vienna, Savoyenstraße 1, Vienna, Austria; 2 Department of Interdisciplinary Life Sciences, Research Institute of Wildlife Ecology, University of Veterinary Medicine Vienna, Savoyenstraße 1, Vienna, Austria; 3 International Flavors & Fragrances Inc., 1515 State Route 36, Union Beach, NJ USA

**Keywords:** Chemical biology, Ecology, Evolution, Zoology, Ecology

## Abstract

The major urinary proteins (MUPs) of house mice (*Mus musculus*) bind and stabilize the release of pheromones and other volatile organic compounds (VOCs) from urinary scent marks, which mediate chemical communication. Social status influences MUP and VOC excretion, and the urinary scent of dominant males is attractive to females. Urinary pheromones influence the sexual behavior and physiology of conspecifics, and yet it is not known whether they also affect reproductive success. We monitored the excretion of urinary protein and VOCs of wild-derived house mice living in large seminatural enclosures to compare the sexes and to test how these compounds correlate with reproductive success. Among males, urinary protein concentration and VOC expression correlated with reproductive success and social status. Territorial dominance also correlated with reproductive success in both sexes; but among females, no urinary compounds were found to correlate with social status or reproductive success. We found several differences in the urinary protein and volatile pheromones of mice in standard cages versus seminatural enclosures, which raises caveats for conventional laboratory studies. These findings provide novel evidence for chemical signals that correlate with male reproductive success of house mice living in competitive conditions.

## Introduction

Male house mice scent-mark their territories with urine and they excrete several compounds often proposed to enhance mating and reproductive success^1–4^. Males produce large quantities of protein in their urine, mainly composed of major urinary proteins (MUPs)^5,6^. MUPs bind and stabilize the release of volatile organic compounds (VOCs) from urinary scent marks^7,8^. These VOCs include the male pheromones, α- and β-farnesene, 2-s-butyl-4, 5-dihydrothiazole (SBT), 3,4-dehydro-exo-brevicomin (DHB), and 6-hydroxy-6-methyl-3-heptanone (HMH), which trigger changes in female sexual development, physiology and behavior^1,9,10^. Trimethylamine (TMA) is a sexually dimorphic VOC that is highly expressed in males. Interestingly, it is attractive to mice at normal levels, but aversive at high levels in urine^11^. MUP proteoforms can act as pheromones as well as transporters, and MUP20 (darcin) is a predominantly male-expressed urinary protein that elicits place preferences and spatial learning in female mice^12^. Females detect MUPs in male urine by upregulating VNO receptor expression during estrus^13^, and exposure to a combination of volatile male pheromones (SBT, DHB, and HMH) induces female olfactory preferences for these compounds and accelerates puberty^1,14^ (but see^15^). Most mammalian studies on female responses to male pheromones examined domesticated mouse strains in artificial laboratory conditions, and focused on female sexual development, estrous cycling, lordosis, or pregnancy block. It is still not known whether male pheromones influence reproductive success. Our first goal was to test whether these pheromones influence the reproductive success of wild-derived male house mice in seminatural conditions.

One way that chemical signals are often suggested to influence reproductive success is by providing a reliable indicator of social status^16^. Wild house mice are highly territorial, and dominant, territorial males have higher reproductive success than non-territorial subordinates^17^. Males that are socially aggressive have higher urinary protein concentrations^18,19^ (but see^20^), and produce higher intensities of particular volatile pheromones (DHB, HMH, SBT, and α/β-farnesene) than submissive males^10,21^. Social defeat can result in decreased expression^21^. Estrous females prefer the urinary scent of aggressive 'dominant' males over submissive 'subordinate' males^22^. An important caveat to these studies is that most were conducted with laboratory mice, and male social status was assessed using the outcomes of brief, dyadic agonistic interactions in the laboratory; a proxy that does not necessarily predict social status in more natural social conditions. Indeed, one study found that this proxy did not correlate with social status of group-housed male mice^23^. A recent study on wild-derived house mice living in seminatural conditions found that once males acquired a territory and became socially dominant, they increased the production of some (MUP20 and HMH), but not other pheromones (e.g. SBT, DHB, farnesene), whereas males did not reduce pheromone excretion after they became subordinates^24^. Estrous females were more attracted to the urinary scent of dominant, territorial males than subordinates, and variation in protein concentration of male urine had no effect on female preferences when male social status was controlled. This study confirmed that male pheromone expression is context- and status-dependent and that estrous females are more attracted to the scent of dominant than subordinate males, but unlike studies on social defeat in the laboratory, subordinate males did not reduce pheromone excretion in naturalistic conditions.

Studies are also needed to compare the expression of chemical signals between the sexes in more natural social contexts, and test for compounds that influence female reproductive success. Putative pheromones in female urine include 2-heptanone^25^, 2,5-dimethylpyrazine^25^, and isobutylamine (IBA)^26^, which have been reported to signal estrus^27,28^, attract males^28^, and delay puberty in juvenile females^29,30^ (though the opposite effect is reported for IBA^26^). Yet the pheromones of female mice remain under-investigated, and there have been no studies on female VOCs in seminatural conditions to our knowledge. Two studies investigated female MUP excretion in seminatural conditions, and one found that female MUP excretion was positively correlated with aggressive behaviors^31^, whereas another found no such relationship^24^. Both studies found that the large sex difference in urinary protein concentration reported in standard laboratory conditions was significantly lower when mice live in seminatural conditions due to increased female urinary protein excretion^24,31^. Therefore, our second aim was to compare the production of volatile and non-volatile urinary compounds between the sexes, and test whether these compounds are regulated depending upon social status or correlate with reproductive success.

We conducted our study on wild-derived house mice (F3 from wild-trapped *M. musculus* *musculus*) in seminatural conditions. We released mice into four indoor enclosures (9 m × 4 m each; Supplementary  Fig. S1) for 16 weeks and recorded their social behavior. Urine was collected at 4-week intervals throughout the experiment. We measured urinary protein and used gas chromatography coupled with mass spectrometry (GC–MS) to quantify VOCs at multiple time points, allowing us to compare pheromone expression before and during the seminatural enclosure phase. We expected that upregulation and excretion of MUPs and volatile pheromones would influence male reproductive success, and that the regulation of these compounds would depend upon their social status^24^. Furthermore, we expected that the degree of sexual dimorphism in urinary compounds would change in competitive conditions due to socially-mediated effects on the chemical signals of both sexes^24,31–34^, but we did not expect females to regulate protein excretion depending upon their social status^24^ or to correlate with reproductive success^31^.

## Results

### Reproductive success

**Figure 1:**
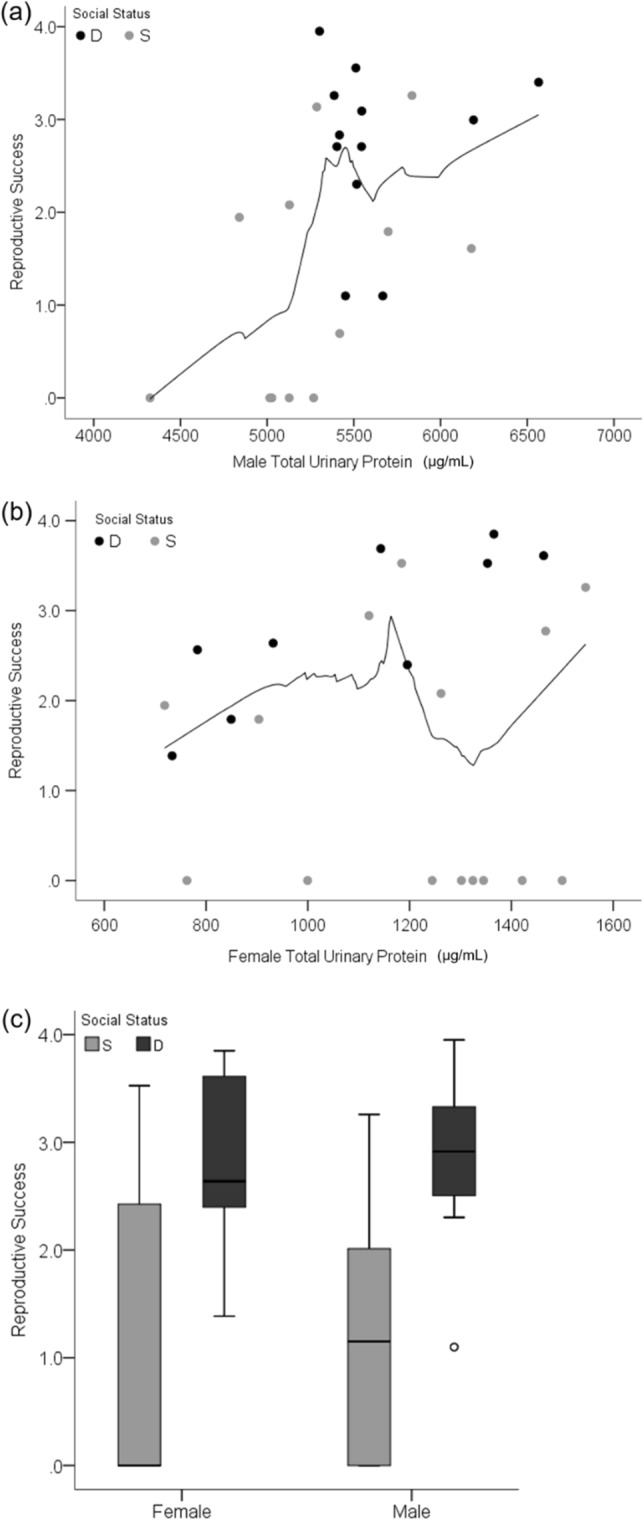
Reproductive success in relation to urinary protein and social status. Scatterplots show the total urinary protein concentration of males (**a**) and females (**b**) in relation to reproductive success. The boxplot (**c**) shows female and male social status in relation to reproductive success. Light gray coloration of data points and boxes indicate subordinate (S) social status during the enclosures. Black data points and dark gray boxes indicate dominant (D) social status. The black trend line in the scatterplots shows the loess (local regression) fit for non-parametric data (50% of data points to fit Epanechnikov kernel).

The production of urinary pheromones correlated with male but not female reproductive success (RS; defined in “Materials and methods” section). The most important predictors of male RS were total urinary protein concentration (75%) and social status (69%; Table 1; based on conditional model average sum of weights). The relative importance of age, creatinine, and mass ranged from 23 to 39%; PC ratio (protein:creatinine concentration) was excluded from the model due to collinearity (VIF = 6.97). Total urinary protein concentration during the enclosure phase was positively correlated with RS for males (Spearman R = 0.52, p = 0.01; Fig. 1a), but not females (Fig. 1b). This correlation is explained by the low protein concentration in the urine of non-reproductive males, as it is no longer significant after removing these males from the analysis (R = 0.12, p = 0.62; Supplementary Fig. S2). The median total urinary protein concentration was 5512 µg mL^−1^ and 5028 µg mL^−1^ for reproductive and non-reproductive males, respectively (Wilcoxon rank sum test *W* = 5, p < 0.001; Supplementary Fig. S2).

**Table 1:** Male reproductive success in response to urinary protein and social status.

Model predictor	Estimate (SEM)	*z*-value	p-value	Sum of weights	No. containing models	Variance inflation factor (VIF)
(Intercept)	− 3.57 (5.88)	0.59	0.55	–	–	–
*Total protein*	*1.47E−3 (5.78E−4)*	*2.54*	*0.011*	*0.75*	*16*	*1.32*
*Social status*	*− 1.10 (0.51)*	*2.05*	*0.04*	*0.69*	*16*	*1.69*
Age	1.40E−2 (8.70E−3)	1.51	0.13	0.39	16	1.49
Creatinine	− 3.30E−3 (2.57E3)	1.21	0.22	0.32	15	1.33
Mass	− 1.71E−1 (1.76E−1)	0.91	0.36	0.23	16	1.69

LME conditional model average for effects of male urine composition, age, mass, and social status during enclosures on RS (natural log transformed (1 + no. of offspring)). Table rows are ordered by predictor importance based on the sum of weights. Significant predictors are italicized. PC ratio omitted due to high VIF value suggesting collinearity (see Supplementary Table S1 for full model).

The most important predictors of female RS were mean body mass (89%) and social status (76%), whereas age, PC ratio, and total protein and creatinine concentration ranged from 14 to 20% (Supplementary Table S1). Female mean body mass during the enclosure was positively correlated to RS (R = 0.57, p = 0.004). When mean body mass during the enclosure is replaced with initial body mass as a model predictor, the relative influence of social status on female RS is 94%; all other variables ranged from 14 to 34% with initial body mass at 23% (Supplementary Table S1). For both sexes, dominant individuals (male = 12; female = 9) accounted for the majority of reproduction compared to subordinates (male = 12; female = 15; Welch’s t-test post hoc male p = 0.006, female p = 0.01; Fig. 1c). Reproduction in the enclosures resulted in 306 offspring from 51 litters (multiple paternity in 69%; Supplementary Table S2). Mate fidelity was 29% and 8% for males and females, respectively. The non-reproductive mice were all subordinates (male = 5; female = 8).

**Figure 2:**
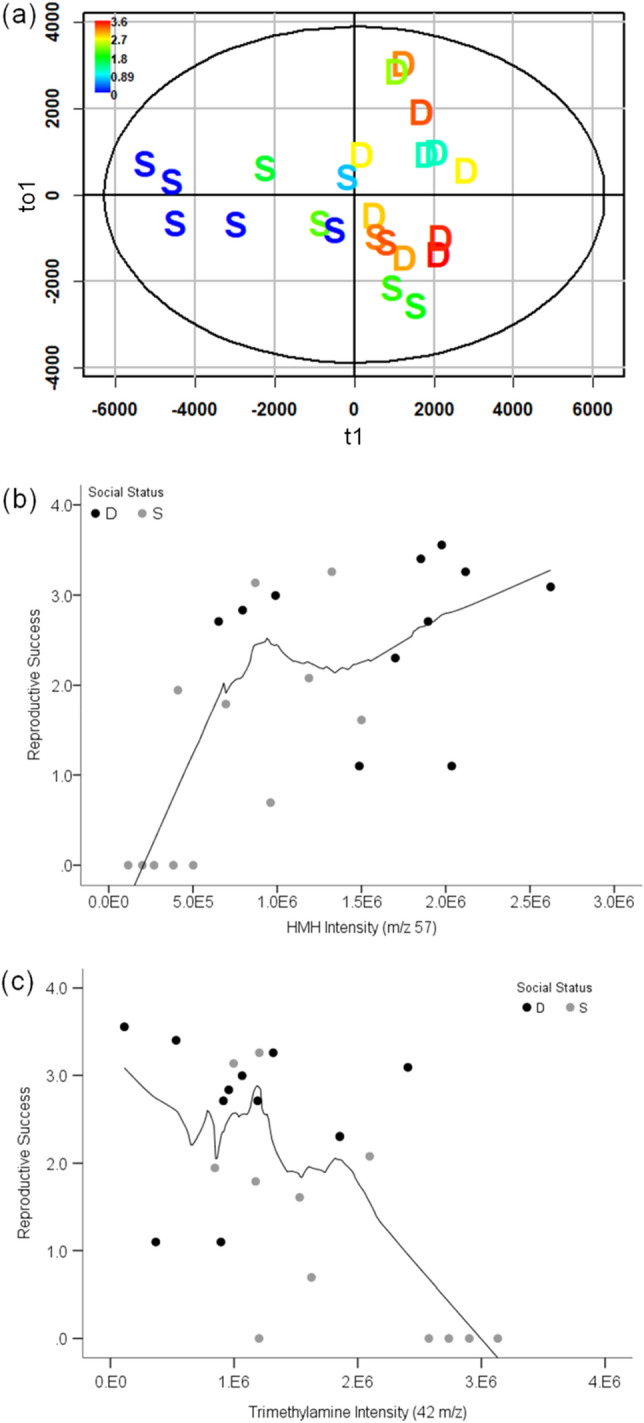
Male reproductive success in relation to VOC expression. OPLS scores plot of reproductive success based on candidate MS-data derived from denatured male urine collected during the enclosure phase (**a**). The x-axis of the scores plot is the predictive component (t1) of the RS response variable. The y-axis is the first orthogonal component (to1). Data points for dominant and subordinates are labeled D and S, respectively. Coloration of the data points indicates the range of RS measured for males; high and low RS range from red to blue, respectively. The Spearman rank correlation of HMH and Trimethylamine expression (**b** and **c**, respectively) with RS shown for intact urine from dominant (black) and subordinate (gray) males during the enclosure phase. The black trend line in the scatterplots shows the loess fit for non-parametric data (50% of data points to fit Epanechnikov kernel).

Male urinary VOC expression during the enclosure phase also correlated with male reproductive success. The explained variance (*R*^2^*Y*) and cross validation score (*Q*^2^) of orthogonal partial least-squares (OPLS) models showed a significant correlation between RS and VOC expression of denatured and intact urine (Fig. 2a; denatured: *R*^2^*Y* = 0.54, *Q*^2^ = 0.46; intact: *R*^2^*Y* = 0.51, *Q*^2^ = 0.39). Two specific urinary volatiles, HMH and TMA, correlated with male RS. In intact urine, peaks corresponding to HMH expression during enclosures were positively correlated to RS (Fig. 2b; R = 0.63, p_adj_ < 0.004), but this correlation is weak in denatured urine (R = 0.47, p_adj_ = 0.02 (n.s.)). We also confirmed that minor ions of HMH in intact urine were correlated with male RS (8 HMH peaks: R > 0.61, p_adj_ < 0.004). TMA was negatively correlated with RS during the enclosure phase, regardless of protein conformation (Fig. 2c; intact: R  = − 0.59, p_adj_ < 0.004; denatured: R  = − 0.55, p_adj_ < 0.008). After omitting non-reproductive males, the correlations between reproductive male RS and both HMH and TMA expression were not significant (HMH: R = 0.23, p = 0.36; TMA: R = − 0.12, p = 0.62; Supplementary Fig. S2). Significant differences in HMH and TMA expression were observed when comparing reproductive and non-reproductive males (Wilcoxon rank sum test p < 0.003 for both VOCs; Supplementary Fig. S2).

Male RS was correlated with both pheromone excretion and social status, and therefore, we examined dominants and subordinates separately and re-ran OPLS models to isolate the effect of VOC expression on reproduction. VOC expression and RS did not correlate among dominant males (OPLS model *Q*^2^ < 0, p > 0.05), whereas the VOC expression of intact urine from subordinate males was strongly correlated with RS, and to a lesser degree in denatured urine (intact: *R*^2^*Y* = 0.75, *Q*^2^ = 0.64; denatured: *R*^2^*Y* = 0.59, *Q*^2^ = 0.49). We found a correlation of subordinate male RS with HMH expression (R = 0.71, p = 0.01), and a negative correlation with TMA (R = − 0.70, p = 0.01), though neither were significant after Bonferroni adjustment for multiple comparisons (refer to “Materials and methods”).

Among females, we found no significant associations between VOC expression during the enclosure phase and RS (OPLS models: *R*^2^*Y* and *Q*^2^ p > 0.05; Supplementary Table S3). We also examined whether VOC expression before enclosure phase could predict RS, but OPLS models based on the female and both male MS-datasets showed no significant correlations (*R*^2^*Y* and *Q*^2^ p > 0.05).

### Male urinary proteins

**Figure 3:**
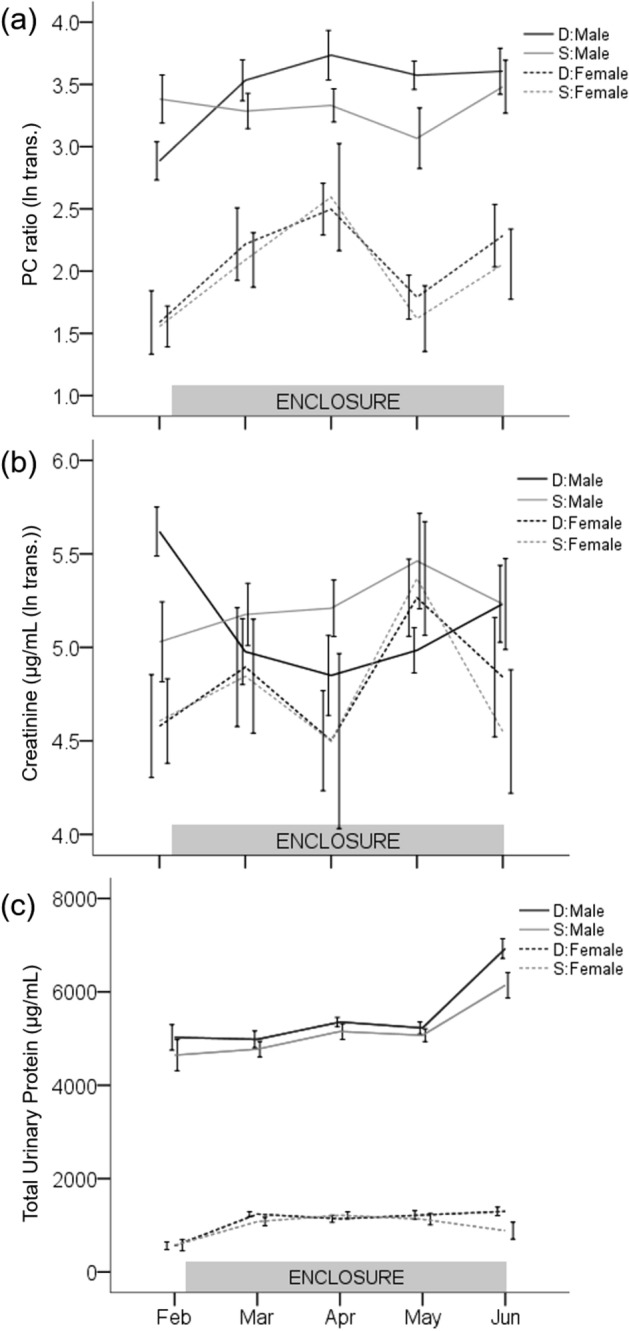
Expression of urinary protein in relation to enclosure phase. Line graphs of PC ratio (ln transformed, **a**), urinary creatinine concentration (µg mL^−1^ (ln transformed, **b**)), and total urinary protein concentration (µg mL^−1^, **c**). Solid and dashed lines indicate males and females, respectively. Black and gray color indicate dominant (D) and subordinate (S) social status, respectively. Note, February is the before enclosure phase measurement; all other time points were during. Error bars are ± 1 SEM.

Male urinary protein excretion in seminatural conditions depended upon social status. Urinary PC ratio (ln transformed) of dominant males significantly increased over time and became higher during the enclosure phase than before (pairwise Tukey post hoc p < 0.04; Supplementary Table S4; Fig. 3a). In contrast, the PC ratio of subordinate males did not vary throughout the experiment (post hoc p > 0.41). Linear mixed effects (LME) modelling reveals that the factors of social status (F_1,76_ = 4.3, p = 0.04), time point (F_4,76_ = 5.3, p < 0.001), and their interaction (F_4,76_ = 3.3, p = 0.01) all had a significant effect on PC ratio. Age had a marginal effect on PC ratio (F_1,76_ = 3.26, p = 0.07), but not body mass (F_1,76_ = 0.5, p = 0.47). Male urinary creatinine concentration (ln transformed), as with PC ratio, changed after release into the enclosures, depending upon social status. Creatinine concentration significantly decreased in dominant male urine during the enclosure phase compared to before (post hoc p < 0.02; Supplementary Table S4; Fig. 3b), whereas PC ratio increased. Urinary creatinine concentration of subordinate males did not vary significantly throughout the experiment (post hoc p > 0.45). The factors of social status (F_1,76_ = 5.4, p = 0.02), time point (F_4,76_ = 4.3, p = 0.004), and their interaction (F_4,76_ = 3.1, p = 0.02) all had significant effects on urinary creatinine concentration, but not age or body mass. The LME model of total urinary protein concentration showed a significant increase over time for both social status groups (F_4,76_ = 15.0, p < 0.001; Supplementary Table S4; Fig. 3c), but was not associated with social status, age, or body mass (all p > 0.12).

We indirectly measured urinary MUP20 production based on liver RNA transcription 14 days after the enclosure phase; however, the LME model average of hepatic *Mup20* gene expression showed no association with social status, RS, or total urinary protein or creatinine concentration in male mice. Predictor importance ranged from 29 to 14%, suggesting a weak, non-significant correlation between *Mup20* transcription and age (29%, R = 0.21, p = 0.35), as well as RS (27%, R = − 0.25, p = 0.28; Supplementary Table S1). Social status was the least important predictor of *Mup20* transcription (14%). A similar pattern was observed when the response variable was absolute hepatic *Mup20* transcription. Predictor importance ranged from 25 to 14% with age and RS as the most important (both 25%; Supplementary Table S1) and social status the least.

### Male urinary VOCs

**Figure 4:**
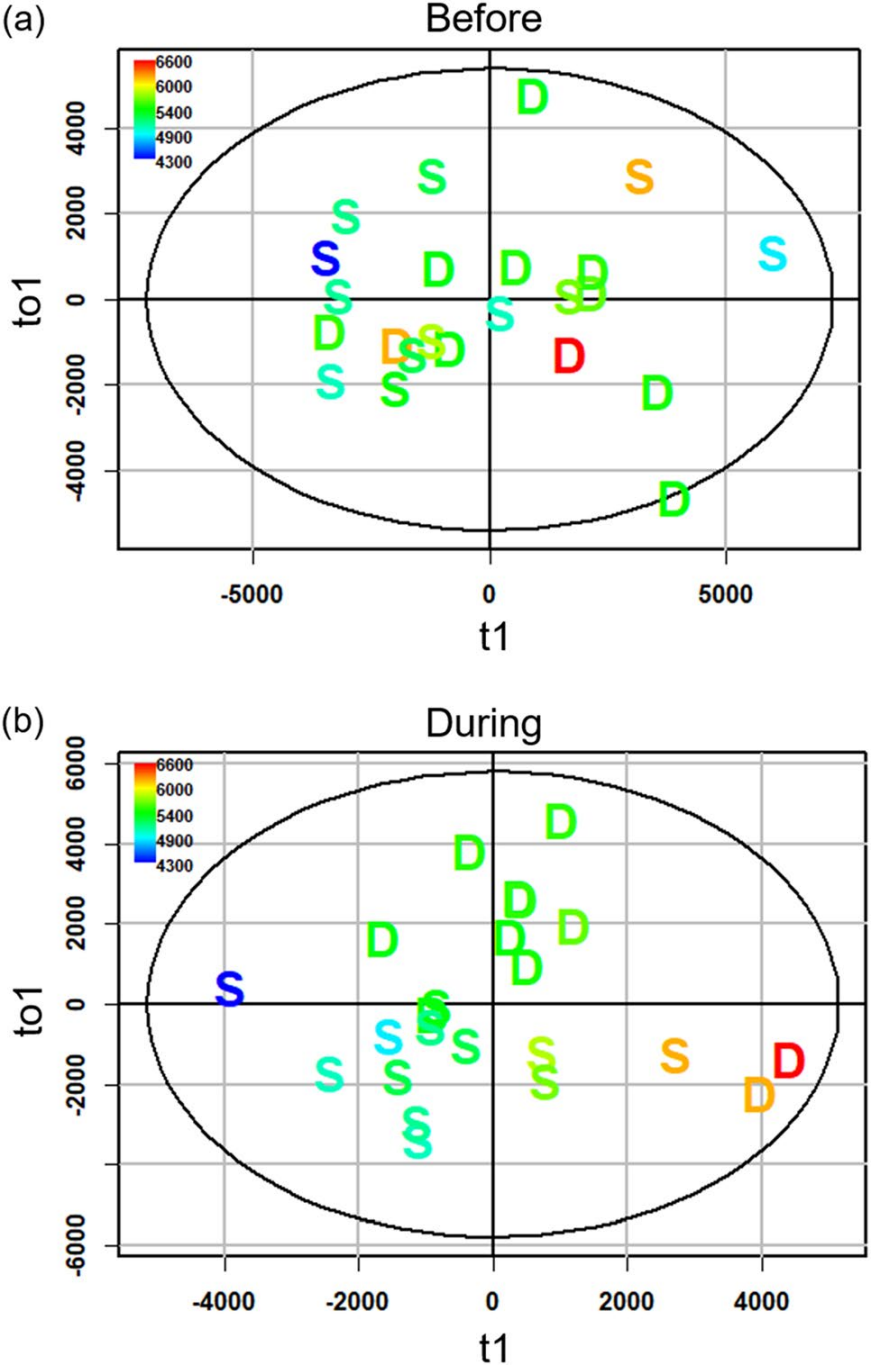
Male VOC expression in relation to urinary protein concentration. OPLS scores plots of total urinary protein concentration based on candidate MS-data derived from denatured male urine (n = 23) collected before (**a**) and during (**b**) the enclosure phase. The x-axis of the scores plot is the predictive component (t1) and the y-axis is the first orthogonal component (to1). Data points for dominant and subordinate males are labelled D and S, respectively. Coloration of the data points indicate the range of urinary protein concentration (µg mL^−1^); high and low concentration range from red to blue, respectively.

We used OPLS models to examine correlations between protein concentration and VOC expression in male urine. Total protein in denatured urine during the enclosures showed a stronger correlation with VOC expression than intact urine both before (denatured: R^2^Y = 0.68, Q^2^ = 0.63; intact: R^2^Y  = 0.40, Q^2^ = 0.22; Fig. 4a) and during the enclosures (denatured: R^2^Y = 0.89, Q^2^  = 0.62; intact: R^2^Y = 0.38, Q^2^  = 0.15; Fig. 4b). Regardless of urinary protein conformation, HMH peaks correlate with protein concentration of urine collected before the enclosures (intact: Pearson R = 0.67, p_adj_ < 3.8E−3; denatured: R = 0.77, p_adj_ < 0.005). Other pre-enclosure correlations between VOCs and urinary protein concentration depended on conformation, including SBT from denatured urine (R = 0.74, p_adj_ < 0.005) and TMA from intact urine (R = 0.21, p_adj_ < 3.8E−3). No peaks correlated with total protein concentration of intact or denatured urine during the enclosures (p_adj_ > 0.003).

**Figure 5:**
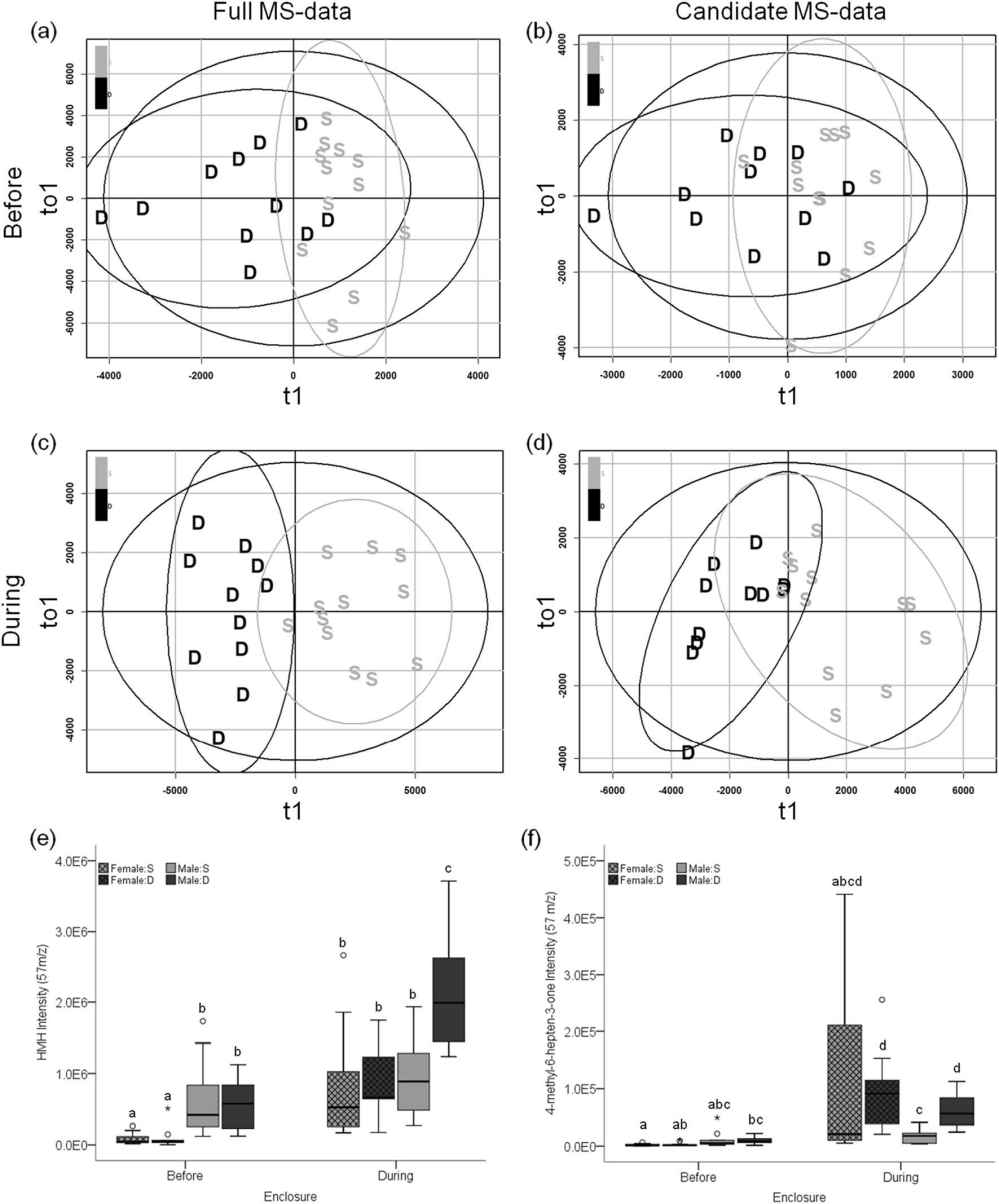
VOC expression in relation to social status. OPLS-DA scores plots of social status before (**a**,**b**) and during the enclosures (**c**,**d**) based on the full MS-data (**a**,**c**) and the candidate MS-data (**b**,**d**) derived from denatured male urine (n = 23).The x-axis of the scores plot is the predictive component (t1) and the y-axis is the first orthogonal component (to1). Data points for dominant (black) and subordinates (light gray) are labelled D and S, respectively. The boxplots show differential expression of HMH and 4-methyl-6-hepten-3-one (**e** and **f**, respectively) in intact urine for dominant (D) and subordinate (S; dark and light gray, respectively) males and females (lattice; n = 24) at both enclosure phases. Different letters above the boxplots denote significant differences.

We tested whether the expression of VOCs in standard conditions predicted male social status during the enclosure phase. The discriminant analysis (OPLS-DA) of VOC expression in denatured urine collected before the enclosure phase did not reliably discriminate males that became dominant during the enclosure phase (Fig. 5a; full MS-data: *R*^2^*Y* = 0.5, *Q*^2^ = − 0.121, misclassification rate (mcr) = 0.17; candidate MS-data: *R*^2^*Y* = 0.311, *Q*^2^ < − 0.01, mcr = 0.26; Fig. 5b). Furthermore, the VOC peak expression and total ion chromatogram (TIC) intensity of pre-enclosure urine did not significantly differ based on the social status the individual obtained during the enclosure phase (Welch’s t-test of TIC: full MS-data p = 0.54; candidate MS-data p = 0.55).

There was a strong association between male social status and urinary VOC expression during the enclosure phase. The OPLS-DA of full MS-data showed robust separation of dominant and subordinate males based on VOC expression of denatured urine collected during the enclosures (*R*^2^*Y* = 0.79, *Q*^2^ = 0.65, mcr = 0.04; Fig. 5c). The denatured urine model of candidate MS-data also showed separation by social status but to a lesser degree (*R*^2^*Y* = 0.62, *Q*^2^ = 0.51, mcr = 0.13; Fig. 5d). The models of intact urine VOC expression also discriminate social status but to a lesser degree than the denatured urine models (intact:full: *R*^2^*Y* = 0.69, *Q*^2^ = 0.41, mcr = 0.17; intact:candidate: *R*^2^*Y* = 0.55, *Q*^2^ = 0.51, mcr = 0.17; Supplementary Table S3). In models of full MS-data, one peak in intact urine and 88 peaks in denatured urine were upregulated in dominant males. The peaks correspond to HMH in denatured urine (mean difference = 1.2E6, Wilcoxon rank-sum post hoc p_adj_ < 5.5E−4; Fig. 5e), and 4-methyl-6-hepten-3-one in both urinary protein conformations (denatured: mean difference = 4.6E4, p_adj_ < 3.5E−4; intact: mean difference = 1.0E4, p_adj_ < 4.4E−4; Fig. 5f). Details for differentiating the spectra of these compounds is in Supplementary Fig. S3. Based on full MS-data, dominant males have a higher TIC intensity than subordinates when comparing denatured urine (mean difference = 1.3E7, p = 0.02), whereas this pattern was not significant for intact urine (mean difference = 7.8E6, p = 0.2). In models of candidate MS-data, peaks that correspond to HMH were upregulated in dominant male intact and denatured urine. The TIC intensity of candidate MS-data did not differ between dominant and subordinate males, regardless of urine conformation (intact urine p = 0.62; denatured urine p = 0.28).

### Female urinary proteins

Female mice showed a significant increase in protein excretion (PC ratio) after being released in the enclosures regardless of their social status (Fig. 3a). We observed a significant effect of time point on female PC ratio (LME: PC ratio (ln transformed): F_4,75_ = 3.3, p = 0.02; Supplementary Table S4), but not for social status, age, body mass, or status:time point interaction (all p > 0.55). Time point also had a strong effect on the LME model of total urinary protein concentration (Fig. 1c; F_4,75_ = 9.9, p < 0.001; Supplementary Table S4). Female mice significantly upregulated total protein concentration and PC ratio during the enclosure phase (Feb-Mar pairwise Tukey post hoc comparison for both D and S p < 0.001; Fig. 3a,c). Age and body mass had a marginal effect on urinary protein concentration in females (age: F_1,75_ = 3.6, p = 0.06; mass: F_1,75_ = 2.8, p = 0.09), but not social status or status:time point interaction (all p > 0.34). The LME of urinary creatinine concentration (ln transformed) was not significantly affected by the model variables (all p > 0.18; Supplementary Table S4), and although stochastic, mean values did not vary significantly between time points (Fig. 3b).

### Female urinary VOCs

Total urinary protein concentration was correlated with VOC expression in denatured female urine, as observed for males but to a lesser extent for female urine (Supplementary Table S3). Total protein concentration of denatured urine collected during the enclosures showed a slightly stronger correlation to VOC expression compared to before the enclosures (before: *R*^2^*Y* = 0.68, *Q*^2^ = 0.44 during: *R*^2^*Y* = 0.71, *Q*^2^ = 0.28). A positive correlation with total protein concentration was observed for 10 peaks before and 2 peaks during the enclosure phase; the VOC(s) to which the peaks correspond were not identified. The OPLS models of female urine examining intact total protein concentration, or PC ratio and creatinine of both intact and denatured urine did not correlate with VOC expression regardless of enclosure phase (p > 0.05; Supplementary Table S3).

Unlike males, VOC expression was not associated with social status in females, regardless of protein conformation and enclosure phase. The OPLS-DA of full MS-data moderately discriminate social status with low predictive ability in denatured female urine (*R*^2^*Y* = 0.52, *Q*^2^ = 0.37, mcr = 0.04), and to a lesser extent in intact urine (*R*^2^*Y* = 0.47, *Q*^2^ = 0.19, mcr = 0.17). For both intact and denatured urine analyses, there were no significant differences in peak intensity based on social status. The TIC intensity was slightly higher for subordinate females during the enclosures, but this difference was not significant (intact:D mean TIC = 2.5E7, intact:S mean TIC = 3.2E7, p = 0.14; denatured:D mean TIC = 2.3E7, denatured:S mean TIC = 2.8E7, p = 0.36). The OPLS-DA of denatured female urine before enclosure phase was not related to social status (*R*^2^*Y* = 0.46, *Q*^2^ < 0.01, mcr = 0.20). There were no expression differences in specific peaks and females that became subordinate during enclosures showed a slightly higher TIC intensity than dominants, though this difference was not significant (before:S mean TIC = 1.3E7, before:D mean TIC = 1.2E7, p = 0.65). With regard to specific female pheromones, the peaks corresponding to 2-heptanone did not correlate with female RS, social status, or urinary protein excretion (*R*^2^*Y* and *Q*^2^ p > 0.05; Supplementary Table S3). Two other female pheromones, IBA and 2,5-dimethylpyrazine, were not detected in any samples.

### Sexual dimorphism of chemosensory signals

Total urinary protein concentration and PC ratio increased significantly during the enclosure phase in both sexes (generalized mixed model (GLMM)); protein concentration Χ^2^ = 28.1, φ = 0.77, p < 0.001; PC ratio Χ^2^ = 28.6, φ = 0.77, p < 0.001; creatinine concentration Χ^2^ = 4.6, φ = 0.31, p = 0.3 (n.s.); Supplementary Table S5). Overall, the mean values of PC ratio and both protein and creatinine concentration were significantly greater for males than females (all p < 0.001). There was a significant sex:housing interaction on urinary protein (Χ^2^ = 43.8, φ = 0.96, p < 0.001) and creatinine concentration (Χ^2^ = 9.1, φ = 0.44, p = 0.002), and a marginal effect on PC ratio (Χ^2^ = 3.7, φ = 0.28, p = 0.053). The interaction result indicates greater sex differences in protein concentration in standard housing conditions (M:F ratio = 8.5; Supplementary Table S5) compared to seminatural enclosure conditions (M:F ratio = 5). Similarly, the degree of sexual dimorphism in urinary creatinine decreased from before (M:F ratio = 1.7) to during enclosure phase (M:F ratio = 1).

Sexual dimorphism in urinary volatiles was discernible after controlling for protein conformation and enclosure phase. OPLS-DA of intact urine better discriminate the sexes before rather than during enclosures (before: *R*^2^*Y* = 0.87, *Q*^2^ = 0.62, mcr = 0.04; during: *R*^2^*Y* = 0.82, Q^2^  = 0.7, mcr = 0.09; Supplementary Table S3). The expression of 82 peaks representing IT, SBT, TMA, and HMH (Fig. 5e) showed a male bias in pre-enclosure intact urine. During the enclosures, we observed a sex-biased expression of 74 peaks (female:male bias 8:66) in intact urine. Peaks representing TMA and SBT were upregulated in males during the enclosure phase, while females upregulated 4-methyl-6-hepten-3-one, which was also upregulated in the denatured urine of dominant males (Fig. 5f). Male TIC intensity of intact urine was greater than female TIC intensity before (mean difference = 1.7E7, p < 0.001) and during the enclosure phase (mean difference = 1.4E7, p < 0.001). As observed with urinary protein levels, the sexual dimorphism of intact urine TIC intensity was significantly greater before compared to during the enclosure phase (M:F before = 2.1; M:F during = 1.5; Χ^2^ = 11.6, φ = 0.71, p < 0.001; Supplementary Table S5).

OPLS-DA of sexual dimorphism are improved when analyzing denatured versus intact urine. Sex discrimination based on VOC expression of denatured urine is more accurate during than before the enclosure phase (before: *R*^2^*Y* = 0.72, Q^2^  = 0.51, mcr = 0.04; during: *R*^2^*Y* = 0.89, Q^2^  = 0.84, mcr = 0.04; Supplementary Table S3). The expression of 88 peaks representing 4-methyl-6-hepten-3-one, HMH, and TMA showed a male bias in pre-enclosure denatured urine. During the enclosures, we observed male-biased expression of 76 peaks, with upregulations of DHB, IT, SBT, and TMA in denatured urine. Male TIC intensity of denatured urine was greater than female TIC intensity before (mean difference = 2.1E7, p < 0.001) and during the enclosure phase (mean difference = 2.1E7, p < 0.001). Consistent with the intact urine result, the sexual dimorphism of denatured urine TIC intensity significantly decreased during the enclosure phase (M:F before = 2.6; M:F during = 1.8; Χ^2^ = 7.9, φ = 0.59, p = 0.005; Supplementary Table S5).

## Discussion

The most important predictor of male reproductive success in the enclosures was urinary protein concentration, which is mainly composed of MUPs^6^. The intensity of HMH, a volatile male pheromone, was also correlated with male RS, and thus, the production of non-volatile and volatile pheromones both correlated with male RS. HMH is unstable during GC analysis and produces several ions with different peaks (Supplementary Fig. S3)^21^, but we found that the minor ions of HMH were correlated with male RS as well. The second most important predictor of male RS was social status, and social status was associated with differences in the excretion of both urinary protein and VOCs. Therefore, the effects of pheromone production on RS could have been through direct male-male competition, female mate choice, or both. MUP excretion may have deterred rival males from entering dominant males' territories^22^, thereby reducing agonistic interactions and mate-competition. MUP excretion may have attracted females to males' territories, or increased female attraction and sexual receptivity by controlling the release of HMH and other pheromones that influence female reproductive physiology and behavior.

Our findings corroborate results from previous studies on social status in wild-derived mice living in seminatural conditions (i.e., reduced reproductive success of subordinate males^17^ and increased urinary protein and HMH pheromone expression in dominant males^24^). Social status did not correlate with body mass, which also confirms results from a previous study in seminatural conditions on mice from this population^24^, but contrary to a result on group-housed laboratory strains^35^. Males that obtained a territory substantially increased urinary protein excretion within four weeks after release in the enclosures, whereas subordinate males did not show any changes in protein excretion over time. There were no differences in pheromone production between dominant and subordinate males before their release into the enclosures, confirming that acquisition of dominant social status influenced pheromone regulation, rather than *vice versa*^24^. The increased protein excretion of dominant males was revealed only after controlling for urine concentration using creatinine levels (PC ratio), and social status had no effect on the total protein concentration. The rate of creatinine production is reportedly consistent for animals of similar body mass^2^, yet a considerable drop in creatinine concentration in dominant males was found in the present study and in a study on domesticated male mice in social housing^23^. It is not known whether urinary creatinine is used as a signal of social status. Low creatinine concentrations can indicate that dominant males excrete higher volumes of urine per day^36^; however, we found that males had similar urine volumes regardless of social status.

Although we did not measure daily urine production, dominant males that upregulated the excretion of MUPs and VOCs may have also increased their urinary scent mark deposition in the enclosures. Indeed, previous studies found that dominant males produced more urine^37^ and scent marks compared to subordinates^38^; and male scent-marking is correlated with RS when females can select their mates^39^. We also investigated hepatic *Mup20* gene expression of males, as high levels of MUP20 excretion have been found in dominant males^24^, but we found no correlations with social status or RS. This negative result is not definitive, however, because males were not sampled until 14 days after terminating the enclosure phase and differences in protein excretion between dominant and subordinate males have been found to disappear after removal from seminatural conditions (≤ 28 days^24^). Nevertheless, this finding supports a previous study showing that that dominant and subordinate males no longer show differences in MUP expression after being removed from competitive conditions^24^.

Social status also correlated with the intensity of VOCs in male urine, and analyses of the full MS-data were better at discerning dominants from subordinates than the candidate MS-data. This finding indicates that social status affected the expression of several unidentified VOCs in male urine. Some volatile pheromones (HMH and 4-methyl-6-hepten-3-one) were differentially expressed in the urine of dominant males, but others were not (DHB, SBT, and farnesene). The urine of dominant, territorial males was also found to have higher intensities of HMH than subordinates in a previous study of wild-derived mice in seminatural conditions^24^. HMH is androgen-dependent and a female attractant, but only when combined with DHB and SBT^40^. The expression of DHB, SBT and farnesene were not upregulated in dominant males, and these volatile compounds were excreted by all males (before and during the enclosures). Therefore, it is possible that they help to elicit reproductive receptivity in females when combined with other chemosensory compounds to form a multicomponent pheromone^4^. The signaling functions of 4-methyl-6-hepten-3-one are not well-studied, though it has been found to be upregulated (along with DHB and SBT) in the urine of aged males (15–20 mo), and is preferred by females in olfactory assays over the urine of younger adults (3–8 mo^41^). Furthermore, the VOC expression in male urine does not sufficiently discriminate dominants from subordinates before the mice were released into the enclosure, indicating that social status regulates VOC production, and not vice versa.

Because pheromone production (urinary proteins and VOCs) and social status were both correlated with male RS, we investigated their independent effects. Unexpectedly, we found that male VOC expression in intact urine was correlated with RS of subordinate but not dominant males. This finding is largely influenced by low HMH and high TMA expression from non-reproductive subordinate males, since subordinate sires had expression levels similar to some dominant males. Therefore, subordinate males may increase their ability to attract females via HMH expression, despite being non-territorial. We found that the intensity of TMA was highly elevated in subordinate males and it was negatively correlated with male reproductive success. TMA is abundant in the urine of mice and shows ca. 30-fold higher levels in males than females^42^. This amine is a metabolite of gut microbiota^43^, and it is sexually dimorphic because females oxidize it in the liver^11^. Mice show an attraction to urine with normal physiological levels, but an aversion to high TMA concentrations (≥ 1000 mM^11^). TMA is detected by trace amine-associated receptors (TAARs^44,45^), and TAAR5 knockout mice lose their attraction to TMA and to mouse urinary scent^11^. Yet, avoidance of high TMA persists in knockout mice, indicating other receptors are sufficient for aversive responses^46^. TMA has been proposed to influence sex and species-specific recognition in mice^44^, and to function as an aversive allomone (*Mus musculus* excrete 1000-fold higher levels than other *Mus* species, and it is repellant to rats^11^). TMA is an indicator of spoilage and putrefaction of dead and decaying animals (it smells like rotten fish to humans), and it is highly aversive to many species^42^. High urinary TMA provides an indicator of parasitic infection (*Schistosoma*^47^ and *Cryptosporidium*^48^), which may help explain how females discriminate and prefer the scent of healthy over infected males^49,50^. TMA is also elevated in the urine of distressed mice (under stress restraint^51^). Taken together, our results show that high TMA is associated with subordinate social status and low reproductive success. Studies are now needed to experimentally test whether females avoid males having high levels of urinary TMA.

In contrast to males, there was no correlation between female RS and urinary protein or VOC expression. Furthermore, females showed no correlation between social status and total protein concentration (or PC ratio), confirming one previous study^24^ but not another one^31^. There was no correlation between VOC expression and female social status, though we did not detect the urinary pheromones Isobutylamine^28^ and 2,5-dimethylpyrazine^1,52^. We confirmed that female RS was correlated with social status^53^, and also body mass, but the latter was undoubtedly due to gaining weight during pregnancy (initial mass showed no such correlation and several females were visibly pregnant during urine collections). Based on these findings and our behavioral observations, dominant females may have deterred subordinate females from mating through direct agonistic interactions, rather than pheromonal excretion of estrus-inhibitors or mate-attractants in urine. Notably, we did not definitively measure female reproductive state, and periodic fluctuations of urinary compounds coincide with stage of estrus^27,33^ or pregnancy^32,54^. Closely monitoring for such effects in seminatural conditions would increase the frequency of handling the mice, and we opted to minimize disturbances that potentially affect behavior^55^.

We confirmed sex differences in urinary protein and volatile excretion of house mice, and also that these well-established sex differences in standard housing are reduced in competitive, seminatural conditions^24^. We confirmed that baseline levels of urinary PC ratio in standard housing conditions applies to subordinate males, but not to dominant males or females^24^. Furthermore, the degree of sexual dimorphism of VOC expression depended on housing conditions and urinary protein conformation. Our findings suggest that the relatively low variation among males and large sex differences in laboratory studies are artifacts due to artificial conditions.

In contrast to a previous study^24^, total protein concentration of intact urine did not correlate with TIC intensity for either sex during the enclosure phase. However, our statistical analyses differed from this previous study, as OPLS models of urinary protein concentration in relation to VOC expression control for sex, housing condition, and protein conformation. Despite our attempts to minimize confounding factors, we observed inconsistent expression of VOCs associated with RS, social status, urinary protein, housing conditions, and sex differences depending on whether the GC–MS data is derived from intact or denatured urine. Disparities in VOC expression are likely due to the affinity of ligands to the MUP binding cavity despite protein denaturation^8^. These results raise additional caveats for results obtained by studying chemical signals of rodents kept in standard housing conditions.

Our results show that the production of specific pheromones correlated with the reproductive success of wild-derived male house mice living in competitive conditions. Males regulated the production of these chemosensory compounds depending upon their social status. To our knowledge, these findings are the first to describe the relationship between pheromone expression and reproductive success in any mammal. Since urinary protein output and HMH intensity correlated with male but not female reproductive success, our findings help to explain the evolution of sexually dimorphic (male-biased) expression of MUPs and HMH in house mice. Female RS was associated with social status based on agonistic interactions, and though we did not find any correlations with specific urinary chemosensory compounds, this does not mean that we can rule them out. More studies are needed to investigate female pheromones and RS. Future studies are also needed to examine the biochemical pathways and neuro-endocrine mechanisms through which males regulate chemosensory signals and experimentally test whether pheromones affect RS under competitive conditions. Furthermore, chemosensory compounds are found in lachrymal, mammary, salivary, and vaginal secretions of mice^56,57^, and though it would require invasive sampling, future studies are needed to incorporate more of the emanations that mice use for chemical communication. Our results suggest that the 'normal' or 'baseline' levels of pheromones found in the laboratory are not ecologically relevant and are more indicative of studying mice in cages. Therefore, efforts to understand the mechanisms and functions of chemical signals require studying animals under more natural social contexts.

## Materials and methods

### Trapping, breeding animals, and standard housing conditions

Experimental animals (N = 48) were virgin F3 offspring of 17 breeding pairs of wild house mice (*Mus musculus musculus*) trapped at seven locations within a 300 m radius in Vienna, Austria (48°13′14″N; 16°17′00″E). Mice were weaned at 21 ± 1 d, separated from siblings at the age of 35 ± 1 d and housed in standard mouse cages (type IIL, 36.5 × 20.5 × 14 cm, Tecniplast, Germany) containing wooden bedding (ABEDD, Austria), a cardboard roll, cotton nestlets© (ABEDD, Austria), and a plastic nest box (Tecniplast, Germany). Water and food (Altromin rodent diet 1324) were provided ad libitum and temperature was maintained at 22 ± 2 °C. Mice were kept on a 12:12 h light:dark cycle with red lights on at 1500. Wild-derived mice in our colony are often aggressive toward same-sex conspecifics when multiply-housed in cages. Thus, all individuals were singly housed from their weaning date until the start of the experiment. We use these descriptions of housing, diet, and light:dark cycle to define standard housing conditions. At weaning, all animals received an ear-punch for individual identification and to obtain tissue for DNA paternity analyses. The Ethical and Animal Welfare Commission at the University of Veterinary Medicine Vienna approved the experimental protocols (permit no. 02/08/97/2013). We confirm that all experiments and animal handling were performed according to the ethical standards and guidelines outlined by the Ethical and Animal Welfare Commission. All reported procedures conformed to the Animal Research Reporting of in vivo Experiments—ARRIVE guidelines^58^.

### Seminatural enclosure housing conditions

Mice were simultaneously released at the center of one of four indoor seminatural enclosures (Supplementary Fig. S1). Each enclosure consisted of 12 mice (1:1 sex ratio) that were sexually mature at the start of the experiment (mean ± s.d. age of males = 134 d ± 28, females = 133 d ± 26). Mice within an enclosure were non-siblings and males were matched for body mass (maximum difference = 0.9 g). Each enclosure (4 × 9 m) was subdivided into eight compartments (wire-mesh fencing, 40-cm high), which mice could scale, but tended to use as territorial boundaries. Wooden bedding (ABEDD, Austria), plastic nest boxes, water stations, wood wool, and nesting material (paper towels) were provided. Food (Altromin rodent diet 1324) and water were provided ad libitum and temperature was maintained at 22 ± 2 °C. Mice lived in the enclosures for 16 weeks between February–June 2016 with a light:dark cycle, as described for standard housing conditions.

### Behavioral observations

To assess social status, behavioral observations of the mice were conducted three to five days per week for 30 min/day between 1500 and 1700 during the entire period (241 h total observation time, 60.3 h mean time per enclosure). Males received unique fur cuts before release, facilitating identification under red light; females were identified by their unique ear punches. Observers monitored the behavior of mice through observation windows from adjacent rooms to avoid disturbance. They recorded interactions (classified as aggressive, submissive, and investigatory behaviors), the location of the interaction, and the individuals involved. A dominance index for each individual was calculated by dividing the number of aggressive and investigatory interactions by the total number of interactions^17^ (depicted in Eq. 1).

1 $$Dominance \; Index= \frac{Aggressive + Investigatory}{Submissive + Aggressive +Investigatory}$$

Mice obtaining a dominance index ≥ 80% within an enclosure compartment were considered to be dominant; otherwise they were considered subordinate. Adult survival was monitored daily and offspring born in the enclosures were removed upon discovery. Offspring tissue was collected for paternity analyses.

### Urine sampling

For monitoring urinary protein and volatile pheromone production, we conducted five urine collection events from each enclosure population over 16 weeks, at 4-week intervals (outlined in Supplementary Fig. S1). The first collection event was conducted immediately prior to the release of mice into the enclosures, while they were still kept in standard housing conditions (‘before enclosure phase’ sample). Four additional collection events occurred while mice were living in seminatural conditions (‘during enclosure phase’). Urine was collected from mice in metabolic cages (Techniplast, Germany), which minimizes handling stress and fecal contamination. All collections were conducted under red light at the beginning of the dark cycle in the enclosures. Upon excreting > 70 µL of urine, mice were put into individual cages and then simultaneously released back into the enclosure (60 min duration for the entire collection event). Only 2 out of 227 urine collections provided an insufficient volume during the sampling periods. Urine and feces were transferred to separate Eppendorf tubes, immediately frozen, and stored at − 80 °C; handling was the same for each sample to avoid possible freezing and storage biases. An aliquot of urine was collected in a glass GC vial for GC–MS analyses and stored as described.

### Urinary protein measurements

As previously described^24^, total urinary protein concentration (µg mL^−1^) was measured in triplicates using a standard Bradford assay on a 96-well microplate^59^. Triplet values not within a ± 10% range were repeated. We adjusted total urinary protein concentration with creatinine concentration to calculate urinary protein excretion (PC ratio), as this value is expected to account for renal activity and urine dilution (creatinine measured by InVitro: Labor für Veterinärmedizinische Diagnostik & Hygiene GmbH, Vienna, Austria). Some studies report 99% of urinary protein is composed of MUPs^60^, in our wild-derived mice, c.85% of the total protein in urine consists of MUPs^6^, yet proteoform expression varies depending on age^61^ and social conditions^24^. In accordance with other studies on urinary protein^19,23,62^, we report PC ratio, total protein concentration (unadjusted values), and creatinine concentration (µg mL^−1^) as response variables in separate models.

### GC–MS analysis of volatile molecules

Urine samples of 23 males (11 dominant, 12 subordinate) and 24 females (9 dominant, 15 subordinate) were obtained before and during the enclosure phase and analyzed with GC–MS as previously described^63^. The ‘during enclosure’ sample was a pool containing an individual’s urine from all collection events while living in the enclosures (outlined in Supplementary Fig. S1). We examined intact urine and denatured urine (15 μL each) because VOC expression in the headspace has been shown to change depending on urinary protein conformation^64^. Pheromonal MUP ligands are elevated after denaturing protein in the urine, as expected, and we denatured urine protein with guanadine hydrochloride (GdmCl) to assess the intensity of these MUP-bound pheromones^63,64^. We are not aware of any alternative approaches for investigating VOCs bound to MUPs (and we are not aware of any volatile artifacts derived from GdmCl). An aliquot of intact urine was denatured with 20 mg of GdmCl (product # G3272, Sigma-Aldrich, Vienna, Austria) in a 4 mL glass vial. The vial was submerged in a water bath at 37 °C and was equilibrated for 10–15 min. The VOCs in the headspace of the sample were extracted by a 2 cm three-component solid phase microextraction (SPME) fiber (30 μm carboxen, 50 μm divinyl-benzene, polydimethylsiloxane; Supelco Corp., Bellefonte, PA, USA) for 15 min at 37 °C. The urine sample in the vial was agitated using a magnetic stirrer during the equilibration period, but not in the extraction period. The SPME fiber containing the adsorbed compounds was then inserted into the injection port of the GC–MS instrument and desorbed for 1 min at 240 °C. A Supelcowax 10 GC column (30 m × 0.25 mm with 0.50 μm film thickness; Sigma-Aldrich, Vienna, Austria) coupled with a Shimadzu GC–MS QP2010 Plus (Duisburg, Germany) were used to analyze the VOCs collected via SPME. The oven temperature was held at 40 °C for 1 min, then programmed at 6 °C/min to 220 °C with a 9-min hold at this final temperature. The carrier gas was helium at a 38.9 cm/sec linear velocity. The transfer line temperature between GC and MS was 250 °C. Operating parameters for the mass spectrometer were as follows: ion source temperature at 200 °C; electron impact ionization (70 eV); and scanning frequency was 4/s from m/z 41 to m/z 300.

Raw GC–MS spectral files were converted to a registry of “peaks” defined as a single ion (mass/charge or m/z) at a specific GC retention time as well as the intensity of that ion for each sample as previously described^64^. Statistical analyses of differential compound expression were performed on the peak registry dataset (N = 1079 peaks), henceforth referred to as the “full MS-data”. Second, we took a candidate approach by focusing on peaks associated with the following male signaling pheromones: (1) 3,4-dehydro-exo-brevicomin (DHB), (2) farnesene, (3) 4-methyl-6-hepten-3-one, (4) 2-isopropyl-4,5-dihydrothiazole (IT), (5) 2-s-butyl-4,5-dihydrothiazole (SBT), (6) trimethylamine (TMA), and (7) 6-hydroxy-6-methyl-3-heptanone (HMH). Peaks were identified after comparison to a mass spectral library (NIST08) combined with manual interpretation. Our GC–MS method accounts for co-elution of HMH with other volatiles, an issue that has been observed for nonpolar SGE columns^65^. The candidate approach yields a peak registry dataset (N = 39 peaks) referred to by the authors as the “candidate MS-data”. The seven listed pheromones correspond to 39 peaks because the fragmentation of a single molecule during mass spectrometry creates multiple ions (peaks), which are then quantified (e.g. 3 peaks correspond to TMA). The analyses of full and candidate MS-data were largely congruent for male urine. We report the results of candidate MS-data in the results section unless otherwise noted; analyses of full MS-data are detailed in Supplementary Table S3. Notably, there is no method for determining the actual total amounts of MUP ligands (or relative intensities), as some unknown portion still remains bound to denatured MUPs even after repeated extractions^8^. Consequently, negative results for MUP ligands should be treated with caution, especially for urine samples with high MUP concentration.

### *Mup20* gene expression and genetic paternity analyses

We used ddPCR to quantify *Mup20* gene expression in the hepatic tissue (henceforth, italicized *Mup20* refers to nucleic acid molecules whereas MUP20 refers to protein). Due to logistical issues, 14 days elapsed between termination of the enclosure phase and euthanization of the mice. Upon euthanization, the liver was removed and immediately immersed in RNAlater (Qiagen) for 24 h before storage at − 80 °C. The RNA from hepatic tissue (c.25 mg) was extracted with RNeasy Mini Kit (Qiagen) following the manufacturer’s instructions, and concentration was measured with a NanoDrop (Thermo Scientific). Between 1 and 1.5 mg of RNA was reverse-transcribed to cDNA using the High-Capacity cDNA Reverse Transcription Kit (Applied Biosystems). The QX200™ Droplet Digital™ PCR system (Bio-Rad) was used to quantify *Mup20* transcripts (247 bp) relative to a non-variable copy number reference gene (*c-myc*, 82 bp, Accession no. NM_001177354). The 20 μL ddPCR mixture was composed of: (1) 10 μL ddPCR supermix for probes (Bio-Rad), (2) 50 μM *Mup20* forward and reverse primers, (3) 10 μM *Mup20* probe, (4) 50 μM *c-myc* forward and reverse primers, (5) 10 μM *c-myc* probe (see Supplementary Table S6 for primer and probe sequences), and 6) 2 μL of cDNA sample. The mixture and 70 μL of droplet generation oil were placed into the DG8 cartridge and inserted to the droplet generator (Bio-Rad) for droplet formation. Droplets were then transferred to a 96-well PCR plate (Eppendorf). The thermal profile for PCR amplification was an initial denaturation at 95 °C for 10 min, followed by 40 cycles of 94 °C for 30 s and 57 °C for 60 s, 1 cycle of 98 °C for 10 min, and ending at 4 °C; reaction performed by C1000 thermal cycler (Bio-Rad). After amplification, the plate was loaded on the droplet reader (Bio-Rad) and quantified. A no-template reaction was included to control for possible reagent contamination. The ratio of *Mup20* positive droplets to *c-myc* positive droplets was used to calculate *Mup20* gene expression. We also measured absolute gene expression by dividing *Mup20* copy number by amount of RNA (ng) used for reverse-transcription. We did not conduct genetic analyses to assess variation in MUPs because a previous survey on this population of mice detected no individual variation in *Mup* gene sequences and low microsatellite diversity throughout the entire *Mup* gene cluster^66^.

For genetic paternity analyses, DNA was extracted from ear punch samples using a proteinase K⁄ isopropanol protocol^67^ and individuals were genotyped at a minimum of nine and a maximum of 14 microsatellite loci (see Supplementary Table S6 for primer sequences). Amplification by PCR and scoring were done as previously reported^39^. Paternity results were confirmed with a ≥ 95% trio confidence (dam-sire-offspring relationship) using the program CERVUS 3.0.3^68^.

### Statistical analyses

Statistical analyses were performed using the R statistical package^69^ (R Development Core Team version 4.0) and the assumptions of the methods used were first verified.

#### Reproductive success (RS)

The relationship between urinary protein excretion and RS was analyzed with a LME model for each sex (*lme* function, package *nlme*^70^). RS was calculated using a natural log transformation of (1 + the number of offspring produced by an individual). Using RS instead of total number of offspring as a measure of fitness provides a Gaussian distribution of the model residuals and accounts for individuals with zero values (no offspring). Social status and the mean values of total urinary protein concentration, creatinine, PC ratio, age, and body mass during the enclosure phase were included as variables in the model with enclosure as a random factor. Due to collinearity in the models, we calculated the VIF for each variable to determine the level of inter-correlation between them. We removed variables sequentially from the model and recalculated VIFs until all values were < 3^71^. Post hoc analyses were performed with Welch’s t-test for categorical variables, or Spearman rank correlation test for continuous variables.

Due to the number of model variables and uncertainty in model selection, a multi-model inference procedure^72^ was used to estimate the relative explanatory importance of each variable. Models were ranked based on Akaike’s Information Criterion value corrected for small sample sizes (AICc) and coefficients for each variable were calculated based on weighted estimates of retained models (the condition for retaining a model was ∆AICc < 10). The relative importance of a variable was established by summing the Akaike weights of each model in which it was a predictor (i.e. variables with high importance are included in models with low AICc). This multi-model inference procedure was applied to LME models in which RS and *Mup20* gene expression are response variables, allowing us to simultaneously test their multiple potential relationships with age, size, social status, and chemical signaling. We refer to this procedure as model averaging.

#### Urinary protein excretion

Three LME models for each sex were used to examine the relationship between urinary protein excretion (PC ratio, total protein concentration, and creatinine concentration) and social status. Model averaging was not performed because fixed factors were inferred from a previous study^24^. PC ratios and creatinine values were natural log transformed to obtain a Gaussian curve of the model residuals. Social status, time point (urine collection event), and the interaction of social status:time point were used as fixed factors; age and body mass were covariates in each model. Since we repeatedly sampled individuals, we include a random factor of individual ID nested in enclosure. We used the *varIdent* function to account for heteroscedasticity in the social status factor and fit the LME model using the maximum likelihood method. The *anova.lme* function produced F and p values for testing significance of model variables. Pairwise comparison post hoc tests were performed for the interaction social status:time point in each model using the *glht* function with Tukey correction for multiple testing (package *multcomp*^73^).

Sex differences in urinary protein excretion were examined with GLMMs (*glmer* function, package *lme4*^74^) due to non-parametric data comprising repeated measures of sexually dimorphic traits. Sex, housing (standard or enclosure), and the sex:housing interaction as fixed effects on the response variables of PC ratio, total protein concentration, and creatinine concentration. A previous study found a correlation between urinary protein concentration and TIC intensity^24^. Therefore, we included the TIC intensity of intact and denatured urine samples as response variables for a total of five GLMMs. Individual ID nested in enclosure were included as random factors. An inverse link function was used to account for the inverse Gaussian distribution of the urinary protein variables. Wald chi-square tests were used to determine significance of the model effects (function *anova*, package *stats*^69^).

#### Gene expression of *Mup20*

LME models were used to examine the relationship between hepatic expression of *Mup20* and urinary protein excretion, social status, and reproduction. Social status, RS, and the mean values of total urinary protein concentration, creatinine, PC ratio, age, and mass during the enclosure phase were included as variables in the model with enclosure as a random factor. We used *Mup20* gene expression and absolute values (based on initial RNA amount) as response variables in separate models. Corrections for collinearity, model averaging, and post hoc analyses were performed as described for RS.

#### VOC expression and identification of differentiating compounds

We conducted OPLS-DA models on the full MS-data with either sex, enclosure phase, or social status as the categorical covariate. The continuous covariates of reproductive success, total urinary protein concentration, creatinine (ln transformed), and PC ratio (ln transformed) were analyzed with OPLS models. Additional models using candidate MS-data were performed on male GC–MS data only. The relationship between MS-data and a given covariate was analyzed using *opls* function in the package *ropls*^75^. The supervised OPLS-DA performs a sevenfold cross validation based on the latent components. This allows us to visualize groups of mice based on the regression of latent components to the covariate. The variance explained by the model (*R*^2^*Y*) and the predictive ability of the model based on cross validation (*Q*^2^) describe the relationship of the covariate to the MS-data. This method also calculates a value of importance to the projection (VIP) for each peak. A large VIP indicates a strong association between a peak and the model covariate. For OPLS-DA, Wilcoxon rank-sum tests with a Bonferroni adjusted p-value due to multiple comparisons (alpha = 0.05) were used to compare the peaks important for the discriminant analysis (VIP > 1) between the two classes of the covariate. Pearson or Spearman rank (non-Gaussian) correlation tests with Bonferroni adjusted p-values were used to compare the association between the continuous covariate with important VOC peaks (VIP > 1) derived from the OPLS model. Since multiple peaks can relate to a single VOC, we provide the results of the greatest VIP peak associated with the male pheromones in the candidate MS-data. The *R*^2^*Y* and *Q*^2^ model coefficients reported in the results section have a corresponding alpha value of 0.05 unless otherwise noted.

## Supplementary Information

Supplementary Information.

## Data Availability

Upon publication, datasets will be available on Mendeley Data repository or by contacting the corresponding authors.
