# Supplementary material for: Pheromones that correlate with reproductive success in competitive conditions
Source: Sci Rep. 2021 Nov 9;11:21970. doi: 10.1038/s41598-021-01507-9 (PMC8578420; doi:10.1038/s41598-021-01507-9)
Supplement: Supplementary file 1 — Supplementary Information. [file 41598_2021_1507_MOESM1_ESM.pdf]

Pheromones that correlate with reproductive success in competitive conditions

Supplementary Tables S1 to S6

Kenneth C. Luzynski*[1]

Doris Nicolakis[1]

Maria Adelaide Marconi[1]

Sarah M. Zala[1]

Jae Kwak[2,3]

Dustin J. Penn*[1]

[1]**Address:** Department of Interdisciplinary Life Sciences, Konrad Lorenz Institute of Ethology, University of Veterinary Medicine Vienna, Savoyenstraße 1, Vienna, Austria

[2]**Address:** Department of Interdisciplinary Life Sciences, Research Institute of Wildlife Ecology, University of Veterinary Medicine Vienna, Savoyenstraße 1, Vienna, Austria

[3]**Address:** International Flavors & Fragrances Inc., 1515 State Route 36, Union Beach, New Jersey, USA

Supplementary Table S1.a. LME model average for effects of male body condition, urine composition, and social status during enclosure phase on reproductive success (log transformed (1 + no. of offspring)). Significant predictors italicized.

Conditional average of predictors

Model	Predictor	Estimate	Standard error of the mean (SEM)	Adj. SEM	z value	p- value for Z-statistic; Pr(>|z|)	Sum of weights	No. containing models	Variance Inflation Factor (VIF)
Full model	(Intercept)	-3.8922	6.1537	6.3043	0.62	0.537	-	-	-
	tot. protein	*0.0015*	*0.0006*	*0.0006*	*2.56*	*0.010*	*0.76*	*31*	*1.61*
	social statu	*-1.0960*	*0.5214*	*0.5508*	*1.99*	*0.047*	*0.65*	*30*	*1.97*
	age	0.0141	0.0087	0.0093	1.53	0.127	0.39	27	1.49
	creatinine	-0.0036	0.0033	0.0035	1.04	0.297	0.3	25	5.96
	PC ratio	0.0089	0.0313	0.0326	0.27	0.786	0.2	27	6.97
	mass	-0.1789	0.1756	0.1867	0.958	0.338	0.23	24	1.72
PC ratio excluded due to collinearity model	(Intercept)	-3.5668	5.8829	6.0287	0.592	0.554	-	-	-
	tot. protein	*0.0015*	*0.0005*	*0.0006*	*2.542*	*0.011*	*0.75*	*16*	*1.32*
	social statu	*-1.1000*	*0.5074*	*0.5362*	*2.051*	*0.040*	*0.69*	*16*	*1.69*
	age	0.0140	0.0087	0.0093	1.506	0.132	0.39	16	1.49
	creatinine	-0.0033	0.0026	0.0027	1.213	0.225	0.32	15	1.33
	mass	-0.1710	0.1762	0.1871	0.914	0.361	0.23	16	1.69
	PC Ratio	-	-	-	-	-	-	-	-

S1.b. LME model average for effects of female body condition, urine composition, and social status during enclosure phase on reproductive success (log transformed (1+ no. of offspring)). Significant predictors italicized.

Conditional average of predictors

Model	Predictor	Estimate	Standard error of the mean (SEM)	Adj. SEM	z value	p- value for Z-statistic; Pr(>|z|)	Sum of weights	No. containing models	Variance Inflation Factor (VIF)
Full model	#(Intercept)	-1.9236	3.3115	3.4246	0.562	0.574	-	-	-
	mass	*0.2184*	*0.0819*	*0.0872*	*2.505*	*0.012*	*0.89*	*22*	*1.43*
	social statu	*-1.2157*	*0.5324*	*0.5682*	*2.14*	*0.032*	*0.76*	*20*	*1.2*
	age	-0.0090	0.0104	0.0111	0.808	0.419	0.2	12	1.62
	PC ratio	-0.0243	0.0376	0.0402	0.606	0.545	0.17	11	2.06
	tot. protein	0.0004	0.0011	0.0011	0.38	0.704	0.15	10	1.93
	creatinine	-0.0007	0.0024	0.0026	0.291	0.771	0.14	11	2.27
Initial mass model	(Intercept)	3.3782	2.4814	2.5737	1.313	0.189	-	-	-
	social statu	*-1.5983*	*0.5359*	*0.5743*	*2.783*	*0.005*	*0.94*	*26*	*1.09*
	age	-0.0145	0.0103	0.0110	1.309	0.191	0.34	13	1.51
	initial mass	0.1127	0.1160	0.1243	0.907	0.365	0.23	16	1.33
	creatinine	-0.0017	0.0025	0.0027	0.641	0.522	0.18	13	2.27
	tot. protein	-0.0002	0.0012	0.0013	0.142	0.887	0.15	13	2.04
	PC ratio	0.0004	0.0407	0.0435	0.01	0.992	0.14	13	2.05

S1.c. LME model average for effects of male condition, urinary composition, social status, and RS on Mup20 gene expression (copy number)

Conditional average of predictors

Model	Predictor	Estimate	Standard error of the mean (SEM)	Adj. SEM	z value	p- value for Z-statistic; Pr(>|z|)	Sum of weights	No. containing models	Variance Inflation Factor (VIF)
Full model	(Intercept)	118.1794	2188	2293	0.05	0.959	-	-	-
	RS	-165.2637	145.1524	155.91	1.06	0.289	0.27	28	2.7
	PC ratio	15.47034	19.1356	20.27	0.76	0.445	0.21	24	6.4
	age	7.70885	6.35344	6.82	1.13	0.259	0.29	28	2.11
	mass	95.29627	107.6067	116.08	0.82	0.412	0.21	26	1.7
	tot. protein	0.01439	0.37839	0.41	0.04	0.972	0.14	24	2.04
	creatinine	0.91411	3.18231	3.34	0.273	0.784	0.16	23	6.31
	social statu	-44.43978	345.4077	372.68	0.119	0.905	0.14	22	2.11
PC ratio excluded due to collinearity model	(Intercept)	199.90	2239.00	2342	0.085	0.932	-	-	-
	RS	-167.40	145.00	155.70	1.075	0.282	0.28	20	2.51
	age	7.87	6.36	6.83	1.153	0.249	0.31	20	2
	mass	99.25	106.90	115.30	0.861	0.389	0.23	19	1.69
	tot. protein	0.00	0.37	0.40	0.001	0.999	0.15	17	1.98
	creatinine	-0.40	1.71	1.85	0.214	0.831	0.15	17	1.5
	social statu	-57.20	345.10	372.20	0.154	0.878	0.15	16	1.97
	PC ratio	-	-	-	-	-	-	-	-

S1.d. LME model average for effects of male condition, urinary composition, social status, and RS on absolute Mup20 gene expression (copy number: RNA mass in nanograms)

Conditional average of predictors

Model	Predictor	Estimate	Standard error of the mean (SEM)	Adj. SEM	z value	p- value for Z-statistic; Pr(>|z|)	Sum of weights	No. containing models	Variance Inflation Factor (VIF)
Full model	(Intercept)	194.7667	3035	3163	0.062	0.951	-	-	-
	mass	143.113	130.7509	141.19	1.014	0.311	0.25	27	1.7
	RS	-190.0112	173.0035	186.40	1.019	0.308	0.25	26	2.63
	PC ratio	23.0501	28.2229	29.62	0.778	0.436	0.22	24	6.39
	tot. protein	-0.2492	0.4315	0.47	0.533	0.594	0.16	23	2
	age	8.6394	7.8894	8.47	1.019	0.308	0.25	27	2.18
	social statu	78.8965	425.1025	459.03	0.172	0.864	0.14	23	2.09
	creatinine	2.4034	4.4406	4.64	0.518	0.605	0.18	24	6.36
PC ratio excluded due to collinearity model	(Intercept)	370.527	3056	3182	0.116	0.907	-	-	-
	mass	146.3217	130.0832	140.38	1.042	0.297	0.27	19	1.7
	RS	-193.2148	173.6234	187.00	1.033	0.301	0.26	19	2.45
	tot. protein	-0.25922	0.42965	0.47	0.557	0.577	0.17	17	1.95
	age	8.89393	7.88387	8.47	1.05	0.294	0.27	19	2.04
	social statu	61.69757	425.2447	459.04	0.134	0.893	0.15	17	1.96
	creatinine	0.01335	2.13074	2.31	0.006	0.995	0.14	17	1.52
	PC ratio	-	-	-	-	-	-	-	-

Supplementary Table S2: Overview of mouse reproduction during the enclosure phase.		
	**Females**	**Males**
# of Offspring (pups)	306	
# of Litters	51	
% Multiple Paternity Litters	69%	
Mean Litters per Mouse	2.125	2.875
Mean Offspring per Mouse	16.1	19.1
% of Mice having Single Mate Fidelity	8.3%	29%
# Non-Reproductive Mice	8	5
Maximum # Offspring for a Mouse	46	51
Maximum # Litters for a Mouse	5	9

cont.

Supplementary Table S3: Orthogonal partial least squares (OPLS) models of GC-MS data collected from wild-derived mouse urine. OPLS-DA (discriminant analysis) models were used for categorical responding variables. RMSEE refers to the root mean squared error of estimation. The number of predictive and orthogonal components describing the multivariate datasets are in the pre and ort columns, respectively. The alpha value of the variation explained by the responding variable and the predictive ability of the model labelled pR2Y and pQ2, respectively. Significant results are italicized.

Sex (status)	Responding variable	Enclosure phase	GC-MS dataset	Protein conformation	R2X	R2Y	Q2	RMSEE	predictive component	orthogonal component	pR2Y	pQ2	mcr
Male	*RS*	*during*	*candidate*	*denatured*	*0.804*	*0.539*	*0.458*	*0.912*	*1*	*1*	*0.05*	*0.05*	--
Male	*RS*	*during*	*candidate*	*intact*	*0.969*	*0.508*	*0.39*	*0.942*	*1*	*1*	*0.05*	*0.05*	--
Male	RS	during	full	denatured	0.592	0.619	0.418	0.829	1	1	0.15	0.05	--
Male	*RS*	*during*	*full*	*intact*	*0.503*	*0.641*	*0.0314*	*0.805*	*1*	*1*	*0.05*	*0.05*	--
Male (Sub)	*RS*	*during*	*candidate*	*intact*	*0.983*	*0.748*	*0.641*	*0.696*	*1*	*1*	*0.05*	*0.05*	--
Male (Sub)	*RS*	*during*	*candidate*	*denatured*	*0.924*	*0.585*	*0.494*	*0.893*	*1*	*1*	*0.05*	*0.05*	--
Male (Dom)	RS	during	candidate	intact	0.762	0.256	-0.325	0.809	1	1	0.55	0.5	--
Male (Dom)	RS	during	candidate	denatured	0.773	0.591	-0.355	0.6	1	1	0.1	0.5	--
Male	RS	before	candidate	denatured	0.773	0.303	-0.08	1.12	1	1	0.15	0.3	--
Male	RS	before	candidate	intact	0.911	0.242	0.0832	1.17	1	1	0.1	0.05	--
Male	RS	before	full	denatured	0.489	0.458	-0.087	0.989	1	1	0.5	0.4	--
Male	RS	before	full	intact	0.47	0.491	0.0554	0.959	1	1	0.4	0.25	--
Male	*tot. prot.*	*before*	*full*	*denatured*	*0.547*	*0.8*	*0.648*	*513*	*1*	*1*	*0.05*	*0.05*	--
Male	*tot. prot.*	*during*	*full*	*denatured*	*0.73*	*0.889*	*0.481*	*174*	*1*	*3*	*0.05*	*0.05*	--
Male	*tot. prot.*	*before*	*full*	*intact*	*0.769*	*0.973*	*0.602*	*210*	*1*	*5*	*0.05*	*0.05*	--
Male	tot. prot.	during	full	intact	0.534	0.577	0.0648	322	1	1	0.05	0.1	--
Male	creatinine	before	full	denatured	0.517	0.616	0.129	89.5	1	1	0.05	0.15	--
Male	creatinine	during	full	denatured	0.45	0.448	-0.445	0.344	1	1	0.65	0.7	--
Male	creatinine	before	full	intact	0.481	0.534	-311	98.6	1	1	0.35	0.35	--
Male	creatinine	during	full	intact	0.562	0.382	-0.491	78.2	1	1	0.5	0.7	--
Male	PC	before	full	denatured	0.491	0.495	0.0414	0.213	1	1	0.25	0.25	--
Male	PC	during	full	denatured	0.434	0.565	-0.394	0.196	1	1	0.3	0.65	--

cont.

Sex (status)	Responding variable	Enclosure phase	GC-MS dataset	Protein conformation	R2X	R2Y	Q2	RMSEE	predictive component	orthogonal component	pR2Y	pQ2	mcr
Male	PC	before	full	intact	0.472	0.592	0.201	13.4	1	1	0.15	0.05	--
Male	PC	during	full	intact	0.575	0.314	-0.336	11.8	1	1	0.7	0.5	--
Male	*tot. prot.*	*before*	*candidate*	*denatured*	*0.886*	*0.68*	*0.634*	*648*	*1*	*1*	*0.05*	*0.05*	--
Male	*tot. prot.*	*during*	*candidate*	*denatured*	*0.998*	*0.891*	*0.622*	*189*	*1*	*6*	*0.05*	*0.05*	--
Male	*tot. prot.*	*before*	*candidate*	*intact*	*0.919*	*0.399*	*0.215*	*889*	*1*	*1*	*0.05*	*0.05*	--
Male	tot. prot.	during	candidate	intact	0.974	0.308	0.149	411	1	1	0.2	0.1	--
Male	creatinine	before	candidate	denatured	0.829	0.363	0.128	115	1	1	0.05	0.1	--
Male	creatinine	during	candidate	denatured	0.868	0.237	-0.636	86.9	1	1	0.45	1	--
Male	creatinine	before	candidate	intact	0.92	0.269	0.0529	123	1	1	0.1	0.15	--
Male	creatinine	during	candidate	intact	0.973	0.155	-0.205	91.5	1	1	0.35	0.6	--
Male	PC	before	candidate	denatured	0.747	0.375	0.207	16.6	1	1	0.1	0.05	--
Male	PC	during	candidate	denatured	0.694	0.288	-0.534	12	1	1	0.15	0.95	--
Male	PC	before	candidate	intact	0.986	0.329	0.209	17.6	1	2	0.1	0.05	--
Male	PC	during	candidate	intact	0.974	0.138	-0.241	13.2	1	1	0.25	0.75	--
Male	*status*	*during*	*full*	*denatured*	*0.508*	*0.786*	*0.647*	*0.248*	*1*	*1*	*0.05*	*0.05*	*0.043*
Male	*status*	*during*	*full*	*intact*	*0.55*	*0.693*	*0.411*	*0.297*	*1*	*1*	*0.05*	*0.05*	*0.174*
Male	status	before	full	denatured	0.515	0.5	-0.121	0.379	1	1	0.3	0.5	0.174
Male	*status*	*during*	*candidate*	*denatured*	*0.838*	*0.615*	*0.511*	*0.332*	*1*	*1*	*0.05*	*0.05*	*0.13*
Male	*status*	*during*	*candidate*	*intact*	*0.974*	*0.555*	*0.511*	*0.357*	*1*	*1*	*0.05*	*0.05*	*0.174*
Male	status	before	candidate	denatured	0.538	0.311	-0.006	0.445	1	1	0.3	0.5	0.261
Female	RS	during	full	denatured	0.603	0.506	0.22	1.08	1	1	0.3	0.1	--
Female	RS	during	full	intact	0.562	0.507	0.228	1.07	1	1	0.1	0.05	--
Female	RS	before	full	denatured	0.432	0.432	-0.029	1.15	1	1	0.6	0.35	--
Female	RS	before	full	intact	0.285	0.445	-0.114	1.14	1	1	0.7	0.4	--
Female	*tot. prot.*	*before*	*full*	*denatured*	*0.471*	*0.676*	*0.438*	*190*	*1*	*1*	*0.05*	*0.05*	--
Female	*tot. prot.*	*during*	*full*	*denatured*	*0.563*	*0.707*	*0.284*	*150*	*1*	*1*	*0.05*	*0.05*	--
Female	tot. prot.	before	full	intact	0.375	0.665	0.391	193	1	1	0.15	0.05	--
Female	tot. prot.	during	full	intact	0.547	0.573	-0.151	181	1	1	0.05	0.5	--

**Sex (status)**	**Responding variable**	**Enclosure phase**	**GC-MS dataset**	**Protein conformation**	**R2X**	**R2Y**	**Q2**	**RMSEE**	**predictive component**	**orthogonal component**	**pR2Y**	**pQ2**	**mcr**
Female	creatinine	before	full	denatured	0.436	0.511	-0.258	0.652	1	1	0.35	0.55	--
Female	creatinine	before	full	intact	0.327	0.663	-0.25	0.541	1	1	0.05	0.55	--
Female	creatinine	during	full	denatured	0.577	0.637	0.236	0.367	1	1	0.05	0.1	--
Female	creatinine	during	full	intact	0.573	0.564	0.23	0.402	1	1	0.05	0.1	--
Female	PC	before	full	denatured	0.442	0.532	-0.167	0.566	1	1	0.35	0.55	--
Female	PC	before	full	intact	0.318	0.57	-0.243	0.543	1	1	0.45	0.5	--
Female	PC	during	full	denatured	0.578	0.569	0.174	0.151	1	1	0.15	0.15	--
Female	PC	during	full	intact	0.554	0.491	0.0587	0.165	1	1	0.2	0.15	--
Female	status	during	full	denatured	0.597	0.522	0.371	0.358	1	1	0.15	0.05	0.042
Female	status	during	full	intact	0.414	0.472	0.193	0.376	1	1	0.25	0.2	0.167
Female	status	before	full	denatured	0.46	0.464	0.0025	0.379	1	1	0.4	0.25	0.208
Both	*sex*	*before*	*full*	*intact*	*0.503*	*0.866*	*0.624*	*0.189*	*1*	*1*	*0.05*	*0.05*	*0.043*
Both	*sex*	*during*	*full*	*intact*	*0.355*	*0.824*	*0.695*	*0.217*	*1*	*1*	*0.05*	*0.05*	*0.085*
Both	*sex*	*before*	*full*	*denatured*	*0.584*	*0.724*	*0.51*	*0.271*	*1*	*1*	*0.05*	*0.05*	*0.043*
Both	*sex*	*during*	*full*	*denatured*	*0.676*	*0.887*	*0.838*	*0.176*	*1*	*2*	*0.05*	*0.05*	*0.043*
Male (sires)	RS; 0-omitted	during	full	intact	0.353	0.605	-1.6	0.589	1	1	0.2	1.05	--
Male (sires)	RS; 0-omitted	during	candidate	intact	0.944	0.089	-0.776	0.894	1	1	0.8	1.05	--
Male (sires)	RS; 0-omitted	during	full	denatured	0.459	0.429	-0.791	0.708	1	1	0.95	0.95	--
Male (sires)	RS; 0-omitted	during	candidate	denatured	0.842	0.105	-0.204	0.886	1	1	0.75	0.6	--
Female	status	during	2-Heptanone	denatured	1	0.193	-0.638	0.494	1	1	0.8	0.85	0.375
Female	status	during	2-Heptanone	intact	1	0.226	-0.384	0.484	1	1	0.65	0.65	0.313
Female	RS	during	2-Heptanone	intact	1	0.116	-1	0.795	1	1	0.95	0.95	--

**Supplementary Table S4.a. LME model for effects of male body condition, social status, and enclosure phase on urinary protein excretion (ln transformed)**							
**FULL MODEL (lnPC)**							
**Fixed effect**	**numDF**	**denDF**	**F value**	**p value**			
(Intercept)	*1*	*76*	*18.424029*	*5.15E-05*			
social status	*1*	*76*	*4.2514584*	*4.26E-02*			
time point	*4*	*76*	*5.3303086*	*7.74E-04*			
age	1	76	3.2575502	7.51E-02			
mass	1	76	0.5168945	4.74E-01			
status:time	*4*	*76*	*3.3244921*	*1.45E-02*			
**Post hoc comparison**							
**social status**	**time point**	**least squares mean**	**standard error**	**df**	**Lower confidence limit**	**Upper confidence limit**	**group significant differences**
DOM	Feb	2.66	0.204	3	1.16	4.17	a
SUB	Feb	3.11	0.23	3	1.41	4.81	ab
SUB	Mar	3.12	0.182	3	1.78	4.47	ab
SUB	May	3.16	0.22	3	1.53	4.79	ab
SUB	Apr	3.34	0.165	3	2.12	4.56	ab
DOM	Mar	3.43	0.194	3	2	4.87	ab
DOM	May	3.72	0.171	3	2.45	4.98	b
SUB	Jun	3.72	0.242	3	1.93	5.51	ab
DOM	Apr	3.75	0.177	3	2.44	5.05	b
DOM	Jun	3.92	0.234	3	2.19	5.65	b
**Table S4.b. LME model for effects of male body condition, social status, and enclosure phase on creatinine (ln transformed)**							
**FULL MODEL (lncreatinine)**							
**Fixed effect**	**numDF**	**denDF**	**F value**	**p value**			
(Intercept)	*1*	*76*	*26.757309*	*1.82E-06*			
social status	*1*	*76*	*5.3500762*	*2.34E-02*			
time point	*4*	*76*	*4.2532189*	*3.69E-03*			
age	1	76	2.2187899	1.40E-01			
mass	1	76	0.00625907	9.37E-01			
status:time	*4*	*76*	*3.0501963*	*2.18E-02*			
**Post hoc comparison**							
**social status**	**time point**	**least squares mean**	**standard error**	**df**	**Lower confidence limit**	**Upper confidence limit**	**group significant differences**
DOM	Apr	4.84	0.184	3	3.48	6.2	a
DOM	May	4.87	0.175	3	3.57	6.16	ab
DOM	Jun	5.01	0.239	3	3.24	6.78	ab
DOM	Mar	5.04	0.2	3	3.56	6.52	ab
SUB	Jun	5.06	0.263	3	3.11	7	ab
SUB	Apr	5.21	0.189	3	3.81	6.61	ab
SUB	Feb	5.24	0.248	3	3.4	7.07	ab
SUB	Mar	5.29	0.203	3	3.8	6.79	ab
SUB	May	5.4	0.25	3	3.55	7.24	ab
DOM	Feb	5.79	0.207	3	4.26	7.32	b
**Table S4.c. LME model for effects of male body condition, social status, and enclosure phase on total urinary protein excretion (mg/mL)**							
**FULL MODEL (Tot. Protein)**							
**Fixed effect**	**numDF**	**denDF**	**F value**	**p value**			
(Intercept)	*1*	*76*	*30.180418*	*5.03E-07*			
social status	1	76	2.4132921	1.24E-01			
time point	*4*	*76*	*14.962704*	*4.61E-09*			

cont.

age	1	76	0.3071358	5.81E-01			
mass	1	76	0.9966577	3.21E-01			
status:time	4	76	1.0369144	3.94E-01			
**Post hoc comparison**							
**social status**	**time point**	**least squares mean**	**standard error**	**df**	**Lower confidence limit**	**Upper confidence limit**	**group significant differences**
SUB	Feb	4513	301	3	2290	6737	a
SUB	Mar	4678	254	3	2803	6554	a
DOM	Feb	4932	245	3	3118	6746	ab
DOM	Mar	4933	236	3	3188	6679	a
SUB	May	5176	308	3	2902	7450	ab
SUB	Apr	5199	240	3	3423	6975	ab
DOM	May	5277	212	3	3709	6844	ab
DOM	Apr	5338	219	3	3718	6957	ab
SUB	Jun	6266	322	3	3886	8647	bc
DOM	Jun	7104	279	3	5038	9171	c
**Table S4.d. LME model for effects of female body condition, social status, and enclosure phase on urinary protein excretion (ln transformed)**							
**FULL MODEL (lnPC)**							
**Fixed effect**	**numDF**	**denDF**	**F value**	**p value**			
(Intercept)	*1*	*75*	*5.8899101*	*0.01762908*			
social status	1	75	0.06248231	0.80329812			
time point	*4*	*75*	*3.2603098*	*0.01605545*			
age	1	75	0.36472769	0.54771467			
mass	1	75	0.0366357	0.8487259			
status:time	4	75	0.18114207	0.94749805			
**Post hoc comparison**							
**social status**	**time point**	**least squares mean**	**standard error**	**df**	**Lower confidence limit**	**Upper confidence limit**	**group significant differences**
SUB	Feb	1.43	0.37	3	-1.303	4.16	a
DOM	Feb	1.51	0.355	3	-1.1192	4.13	a
SUB	May	1.67	0.304	3	-0.5761	3.91	a
DOM	May	1.8	0.264	3	-0.1529	3.76	a
SUB	Mar	1.99	0.261	3	0.0604	3.92	a
SUB	Jun	2.1	0.379	3	-0.6985	4.91	a
DOM	Mar	2.16	0.27	3	0.1653	4.16	a
DOM	Jun	2.41	0.36	3	-0.2561	5.07	a
DOM	Apr	2.51	0.219	3	0.8849	4.13	a
SUB	Apr	2.59	0.3	3	0.3676	4.8	a
**Table S4.e. LME model for effects of female body condition, social status, and enclosure phase on total urinary protein excretion (mg/mL)**							
**FULL MODEL (Tot. Protein)**							
**Fixed effect**	**numDF**	**denDF**	**F value**	**p value**			
(Intercept)	1	75	1.9050876	1.72E-01			
social status	1	75	0.5584213	4.57E-01			
time point	*4*	*75*	*9.8882382*	*1.76E-06*			
age	1	75	3.6004874	6.16E-02			
mass	1	75	2.7883777	9.91E-02			
status:time	4	75	1.1543087	3.38E-01			

Post hoc comparison							
**social status**	**time point**	**least squares mean**	**standard error**	**df**	**Lower confidence limit**	**Upper confidence limit**	**group significant differences**
SUB	Feb	613	158.4	3	-558.4	1784	a
DOM	Feb	701	130.6	3	-264.3	1667	a
SUB	Jun	728	164.7	3	-490.1	1945	ab
SUB	May	1088	135.7	3	84.6	2091	ab
DOM	Jun	1102	133.8	3	113	2092	ab
SUB	Apr	1125	135.7	3	121	2128	ab
DOM	May	1151	90.8	3	479.4	1823	ab
DOM	Apr	1185	68.6	3	677.6	1692	b
SUB	Mar	1218	113.6	3	377.6	2058	b
DOM	Mar	1286	87.2	3	641	1931	b

Table S4.f. LME model for effects of female body condition, social status, and enclosure phase on creatinine (ln transformed)

FULL MODEL (lncreatinine)				
**Fixed effect**	**numDF**	**denDF**	**F value**	**p value**
(Intercept)	*1*	*75*	*1.98E+01*	*2.99E-05*
social status	1	75	5.16E-07	9.99E-01
time point	4	75	1.58E+00	1.88E-01
age	1	75	1.70E+00	1.96E-01
mass	1	75	5.09E-01	4.78E-01
status:time	4	75	1.23E-01	9.74E-01

Post hoc comparison							
**social status**	**time point**	**least squares mean**	**standard error**	**df**	**Lower confidence limit**	**Upper confidence limit**	**group significant differences**
SUB	Jun	4.3	0.456	3	0.93	7.67	a
SUB	Apr	4.44	0.363	3	1.76	7.12	a
DOM	Jun	4.49	0.425	3	1.34	7.63	a
DOM	Apr	4.52	0.253	3	2.65	6.39	a
DOM	Feb	4.82	0.419	3	1.73	7.92	a
SUB	Feb	4.83	0.444	3	1.54	8.11	a
DOM	Mar	5	0.313	3	2.68	7.32	a
SUB	Mar	5.12	0.313	3	2.8	7.43	a
DOM	May	5.15	0.309	3	2.87	7.44	a
SUB	May	5.29	0.366	3	2.58	7.99	a

Supplementary Table S5. GLMM of sexual dimorphism of urinary protein excretion and TIC before and during seminatural enclosure conditions. Significant results italicized.

GLMM

**Responding variable**	**Fixed effect**	**Chisq**	**Df**	**phi**	**p-value**
PC ratio	*sex*	*54.5833*	*1*	*1.50808*	*1.49E-13*
	housing	*28.5891*	*1*	*1.09143*	*8.95E-08*
	sex:housing	3.7255	1	0.39399	0.05359
tot. protein (mg/mL)	*sex*	*327.626*	*1*	*3.69474*	*2.20E-16*
	housing	*78.512*	*1*	*1.80868*	*2.20E-16*
	sex:housing	*43.827*	*1*	*1.35134*	*3.59E-11*
creatinine (mg/mL)	*sex*	*5.9239*	*1*	*0.49682*	*0.014937*
	housing	*4.5534*	*1*	*0.43557*	*0.032853*
	sex:housing	*9.1037*	*1*	*0.61589*	*0.002551*
TIC intact urine	*sex*	*44.647*	*1*	*1.39326*	*2.36E-11*
	housing	*95.929*	*1*	*2.04226*	*2.20E-16*
	sex:housing	*11.553*	*1*	*0.70873*	*0.0006763*
TIC denatured urine	*sex*	*54.7255*	*1*	*1.54252*	*1.39E-13*
	housing	*94.8028*	*1*	*2.03024*	*2.20E-16*
	sex:housing	*7.8873*	*1*	*0.5856*	*0.004978*

Post hoc comparison of Sexual Dimorphism

**Responding variable**	**Enclosure Phase**	**Mean (SEM)**			
		**Male**	**Female**	**M:F ratio**	
PC ratio	Before	29.47 (4.17)	6.61 (1.33)	4.46	
	During	35.35 (2.82)	12.27 (1.52)	2.88	
tot. protein	Before	4823.46 (227.97)	566.09 (66.38)	8.52	
	During	5449.72 (98.27)	1163.21 (54.14)	4.96	
creatinine	Before	238.37 (28.71)	137.2 (23.84)	1.74	
	During	212.39 (19.79)	201.54 (24.46)	1.05	
TIC intact	Before	31415775 (1798268)	14691790 (970843)	2.14	
	During	43511481 (2173169)	29730136 (2649367)	1.46	
TIC denatured	Before	33222512 (2786235)	12569412 (917776)	2.64	
	During	47533279 (2801895)	26050682 (2665100)	1.82	

Supplementary Table S6. Reagents used in genetics analyses.

**Purpose**	**Reagent**	**Oligo sequence**
Mup20 ddPCR	*Mup20* forward primer	5-CTGATGGAGCTCTATGGCCG-3
	Mup20 reverse primer	5-GCAGATCACAGAACTTCTTCTTACTGGA-3
	Mup20 probe	5-[FAM]TTTCTCTAACGATTCCATGCTCCTC[BHQ1]-3
reference gene ddPCR	*c-myc* forward primer	5-CGTCAGAGGAGGAACGAGCT-3
	c-myc reverse primer	5-GGGCCTTTTCGTTGTTTTCCA-3
	c-myc probe	5-[HEX]TGCCCTGCGTGACCAGATCC[BHQ1]-3
Paternity microsat01	D1Mit404 forward primer	5-AGGAATAGAAAAATCAGCAAGCC-3
	D1Mit404 reverse primer	5-CCATTGCCCTTGCTTTAGAA-3
Paternity microsat02	D2Mit380 forward primer	5-CCTCAGGTCTGAAATGAGGTG-3
	D2Mit380 reverse primer	5-AATGATGTGCATGTGCGC-3
Paternity microsat03	D1Mit456 forward primer	5-TGGCTTCCACAGGAATGAG-3
	D1Mit456 reverse primer	5-GCCAGTACAGATGCACAGACA-3
Paternity microsat04	D10Mit20 forward primer	5-CACCCTCACACAGATATGCG-3
	D10Mit20 reverse primer	5-GCATTGGGAAGTCCATGAGT-3
Paternity microsat05	D5Mit25 forward primer	5-AACACACCTCCATACTGGTCG-3
	D5Mit25 reverse primer	5-GGCTAACTGAAATTGTTTTGTGC-3
Paternity microsat06	D15Mit16 forward primer	5-AGACTCAGAGGGCAAAATAAAGC-3
	D15Mit16 reverse primer	5-TCGGCTTTTGTCTGTCTGTC-3
Paternity microsat07	D7Mit227 forward primer	5-GAGTCCTCAGCAGATATTACTCAGC-3
	D7Mit227 reverse primer	5-CTGATGTCTCATCATTTGGGG-3
Paternity microsat08	D2Mit252 forward primer	5-CAGTGCCGTGGAGAAGAAGT-3
	D2Mit252 reverse primer	5-AGTCATCAAGAGATTGACATTACACA-3
Paternity microsat09	D9Mit135 forward primer	5-AGGAAAGAGCCAACTCCCAT-3
	D9Mit135 reverse primer	5-TTTCCTCTCCTTTTTGCGTA-3
Paternity microsat10	D11Mit150 forward primer	5-GGTCAGACACTGAGTGAAGATATAGC-3
	D11Mit150 reverse primer	5-TCCTCTGACACCCATAAGTTCA-3
Paternity microsat11	D19Mit39 forward primer	5-GGAGGTCTCAGGAAATATTACTCC-3
	D19Mit39 reverse primer	5-ATTCCTGTGTAAAGGTGGATGG-3
Paternity microsat12	D6Mit138 forward primer	5-GCTCTTATTAATGAAGAAGAAGGAGG-3
	D6Mit138 reverse primer	5-CAAAGAAAGCATTTCAAGACTGC-3
Paternity microsat13	D9Mit34 forward primer	5-ATGTTAAAACATGGGCTGGTG-3
	D9Mit34 reverse primer	5-TGCTTTCTTGTTATTTCATCTACG-3
Paternity microsat14	D17Mit21 forward primer	5-TAACACCAGACATTGACCTC-3
	D17Mit21 reverse primer	5-AGTCTAGATATGTGTCTCCC-3

Pheromones that correlate with reproductive success in competitive conditions

Supplementary Figure S1 and S2

Kenneth C. Luzynski*[1]

Doris Nicolakis[1]

Maria Adelaide Marconi[1]

Sarah M. Zala[1]

Jae Kwak[2,3]

Dustin J. Penn*[1]

[1]**Address:** Department of Interdisciplinary Life Sciences, Konrad Lorenz Institute of Ethology, University of Veterinary Medicine Vienna, Savoyenstraße 1, Vienna, Austria

[2]**Address:** Department of Interdisciplinary Life Sciences, Research Institute of Wildlife Ecology, University of Veterinary Medicine Vienna, Savoyenstraße 1, Vienna, Austria

[3]**Address:** International Flavors & Fragrances Inc., 1515 State Route 36, Union Beach, New Jersey, USA

cont.

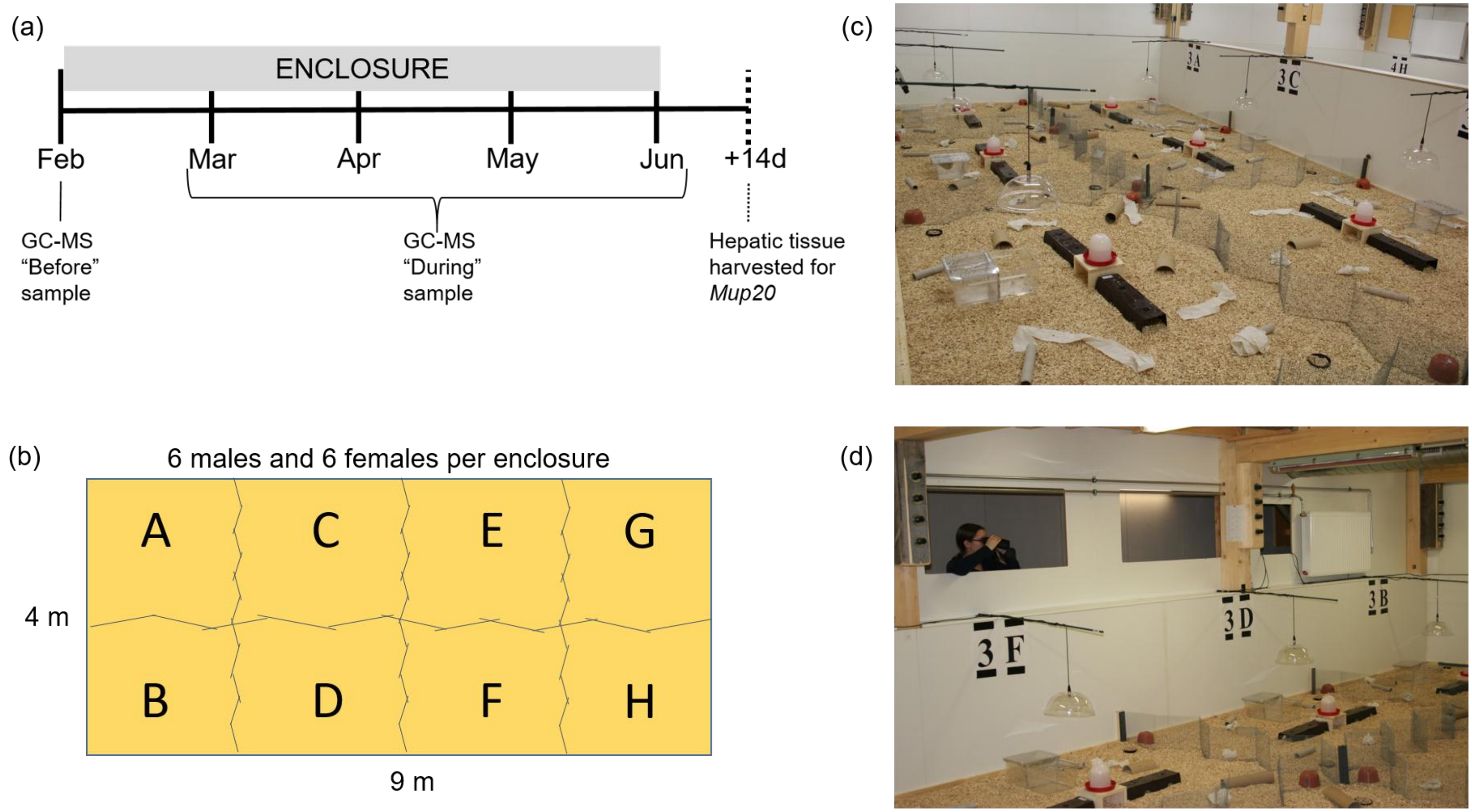

Supplementary Figure S1. Sample collection and enclosure design. Timeline (a; bold black line) of when collection events occurred in relation to when mice were living in enclosures (gray rectangle). Solid hash marks denote the month when urine collection events occurred. Dashed hash marks show when mice were euthanized for harvesting hepatic tissue. GC-MS "During" sample is a pool of the urine collected from mice during the enclosure phase and is denoted with a bracket. Diagram (b) of one of four enclosures viewed from overhead. An enclosure was divided into 8 sectors of equivalent areaa. Capital letters represent sectors within an enclosure separated by gray fencing. Image (c) of an enclosure with nestboxes, nesting material, and food and water stations. Image (d) of observation windows into an enclosure room with an observer. Interactions between mice were recorded after the light period under red light (image lit with white flourescent light to highlight observation windows/method).

cont.

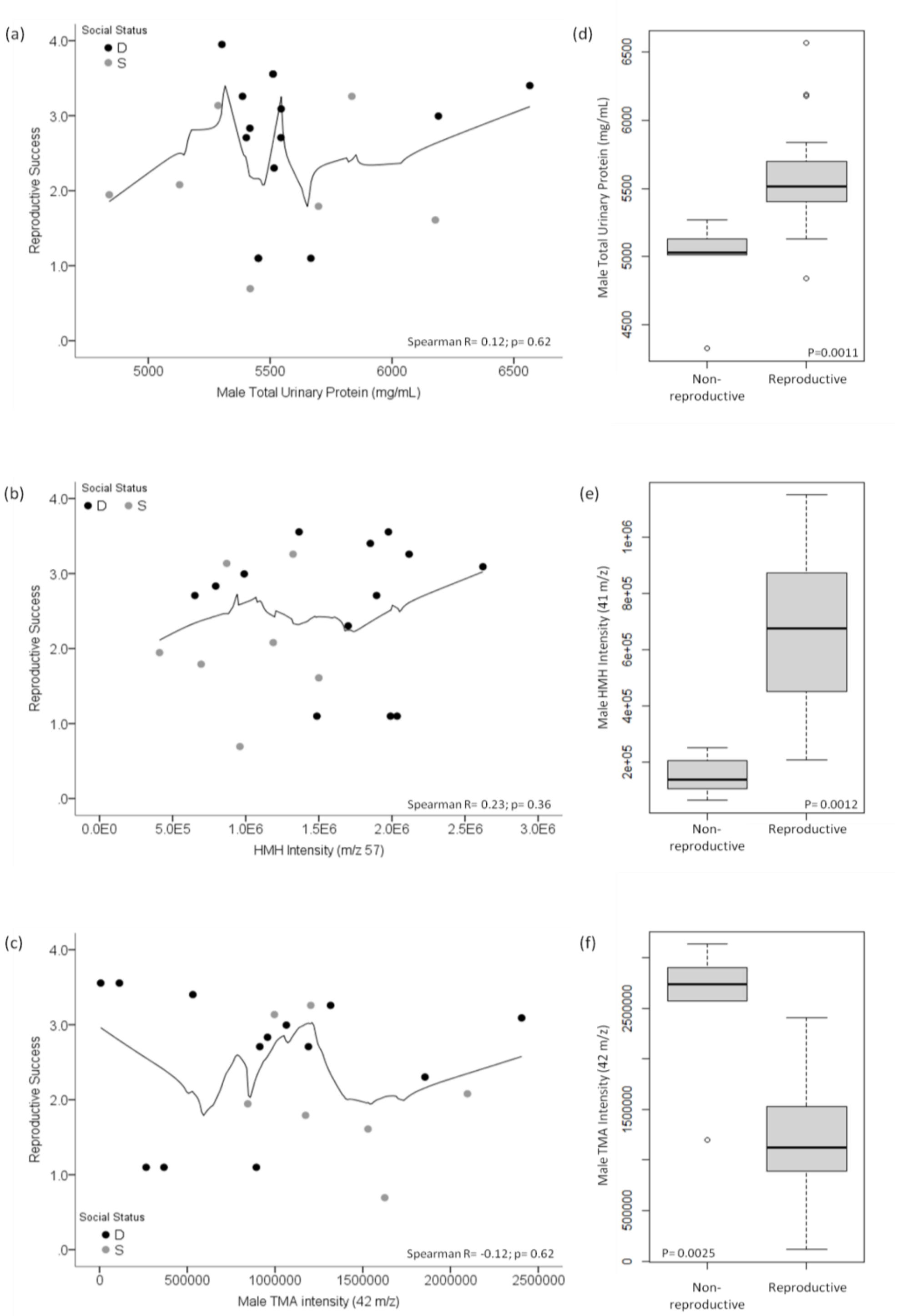

Supplementary Figure S2. Correlations between pheromones and reproductive success weaken when omitting non-reproductive males. Scatterplots of the Spearman correlation of reproductive success with total urinary protein concentration (a), HMH expression (b), and TMA expression (c) of intact urine during the enclosure phase of only reproductive males. Black and Gray colored data points indicate dominant (D) and subordinate (S) males, respectively. The black trend line in the scatterplots shows the loess fit for non-parametric data (50% of data points to fit Epanechnikov kernel). Boxplots show the Wilcoxon rank sum comparison of non-reproductive and reproductive males for total urinary protein concentration (d), HMH expression (e), and TMA expression (f) of intact urine during the enclosure phase. Non-parametric comparisons performed due to low sample size of the non-reproductive class of males.

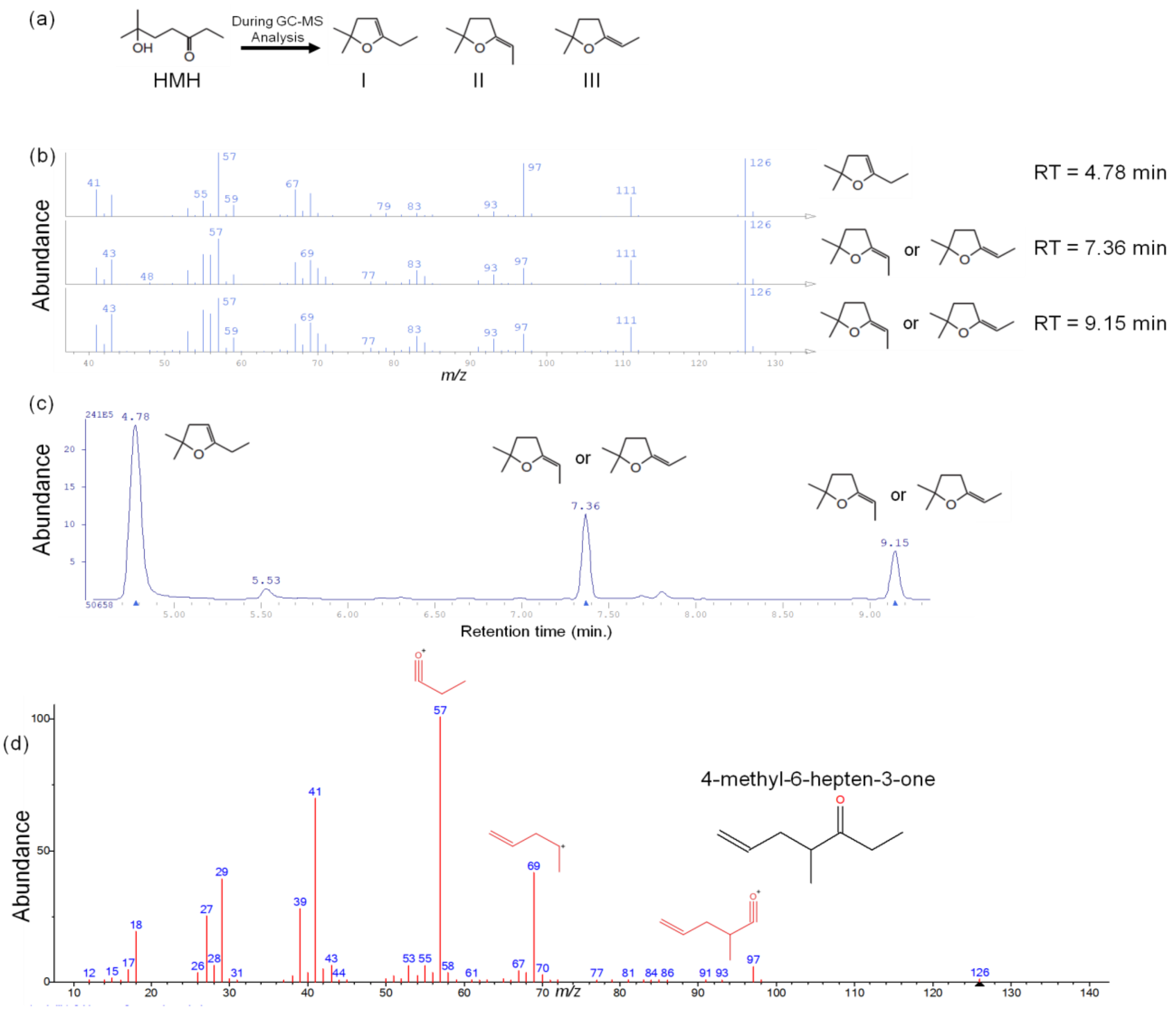

Supplementary Figure S3. A comparison of the spectra of HMH and 4-methyl-6-hepten-3-one. We observed three dehydrated cyclic ethers (skeletal structures I, II, and III) derived from HMH in mouse urine (a). The mass spectra of the three dehydrated cyclic ethers at their given retention time (RT, (b)). The x-axis and blue values above bars indicate m/z for derived ions. The y-axis shows abundance. A graph of the retention time (x-axis) and abundance (y-axis) of the dehydrated cyclic ethers observed in our mouse urine samples (c). The mass spectrum of 4-methyl-6-hepten-3-one along with skeletal structure (d). The x-axis and blue values above bars indicate m/z for derived ions. The skeletal structure of major derived ions are above their respective bars. The three dehydrated cyclic ethers derived from HMH correspond to previously reported peaks (Harvey et al., 1989), confirmed with a synthesized standard (Kwak et al., 2011), and their mass spectra were reported by the group who synthesized HMH (Tashiro et al., 2008). The main difference in the spectra is that the intensity of molecular ion (m/z 126) is stable in the dehydrated cyclic ethers and becomes the base peak in the mass spectrum of HMH (b), whereas this ion is less stable in 4-methyl-6-hepten-3-one and becomes a minor ion (d). Also, the major ions m/z 57 and 69 are observed in 4-methyl-6-hepten-3-one due to alpha cleavages of the carbonyl group and subsequent loss of carbon monoxide, which are not observed to the same extent in HMH. In summary, the identification of three dehydrated cyclic ethers HMH is valid as they were previously confirmed with a synthesized authentic standard and their mass spectra differ from that of 4-methyl-6-heptene-3-one.

References for Supplementary Materials

Harvey, S., Wiesler, D., & Novotny, M. (1989). Formation of cyclic enol ethers from a labile biological precursor: An example of analytical artifacts. Journal of Chromatography B: Biomedical Sciences and Applications, 491(C), 27–36. https://doi.org/10.1016/S0378-4347(00)82816-9

Kwak, J., Josue, J., Faranda, A., Opiekun, M. C., Preti, G., Osada, K., Yamazaki, K., & Beauchamp, G. K. (2011). Butylated Hydroxytoluene Is a Ligand of Urinary Proteins Derived from Female Mice. Chemical Senses, 36, 443–452. https://doi.org/10.1093/chemse/bjr015

Tashiro, T., Osada, K., & Mori, K. (2008). Syntheses of 2-Isopropyl-4,5-dihydrothiazole and 6-Hydroxy-6-methyl-3-heptanone, Pheromone Components of the Male Mouse, Mus musculus. Bioscience, Biotechnology, and Biochemistry, 72(9), 2398–2402. https://doi.org/10.1271/BBB.80293
